# Correlation between crystallographic anisotropy and dendritic orientation selection of binary magnesium alloys

**DOI:** 10.1038/s41598-017-12814-5

**Published:** 2017-10-19

**Authors:** Jinglian Du, Zhipeng Guo, Ang Zhang, Manhong Yang, Mei Li, Shoumei Xiong

**Affiliations:** 1 School of Materials Science and Engineering, Tsinghua University, Beijing, 100084 China; 2 Laboratory for Advanced Materials Processing Technology, Ministry of Education, Tsinghua University, Beijing, 100084 China; 3 Materials Research Department, Research and Innovation Center, Ford Motor Company, MD3182, P.O Box 2053, Dearborn, MI48121 USA

## Abstract

Both synchrotron X-ray tomography and EBSD characterization revealed that the preferred growth directions of magnesium alloy dendrite change as the type and amount of solute elements. Such growth behavior was further investigated by evaluating the orientation-dependent surface energy and the subsequent crystallographic anisotropy *via ab-initio* calculations based on density functional theory and *hcp* lattice structure. It was found that for most binary magnesium alloys, the preferred growth direction of the α-Mg dendrite in the basal plane is always $\langle 11\bar{2}0\rangle$, and independent on either the type or concentration of the additional elements. In non-basal planes, however, the preferred growth direction is highly dependent on the solute concentration. In particular, for Mg-Al alloys, this direction changes from $\langle 11\bar{2}3\rangle$ to $\langle 22\bar{4}5\rangle$ as the Al-concentration increased, and for Mg-Zn alloys, this direction changes from $\langle 11\bar{2}3\rangle$ to $\langle 22\bar{4}5\rangle$ or $\langle 11\bar{2}2\rangle$ as the Zn-content varied. Our results provide a better understanding on the dendritic orientation selection and morphology transition of magnesium alloys at the atomic level.

## Introduction

Magnesium alloy is one of the most promising structural and functional materials due to lightweight, efficient recyclability, high specific strength and stiffness^1–3^. These outstanding properties are highly dependent on the microstructures formed during solidification, of which the primary phase or the α-Mg dendrite, together with the precipitated phases and impurity segregation, exert considerable effects on the ultimate performance of the final products^4–8^. The dendritic microstructure forms according to a first order phase transition driven by non-equilibrium thermodynamic and kinetics at the solid-liquid interface^9–13^. The dendrite prefers to grow along certain directions due to crystallographic anisotropy induced by specific atomic stacking sequence, e.g. *hcp* (hexagonal-close-packed) structure for magnesium, and thus exhibits diverse patterns for morphology transition^14–16^.

Extensive effort has been devoted to understanding the dendritic microstructure formation during solidification of metallic alloys, in particular those with cubic symmetrical lattice structure^17–20^. An *fcc* (face-centered-cubic) structure aluminum alloy dendrite usually grow along $\langle 100\rangle$ ^21^. However, it was found that the growth orientation of Al-Zn alloy dendrite changed from $\langle 100\rangle$ to $\langle 110\rangle$ as the zinc content increased, i.e. the so-called dendrite orientation transition (DOT) occurred^22–24^. For magnesium alloy dendrite with an *hcp* lattice structure, different preferred growth directions with complex dendritic morphology have been reported^25–28^. Edmunds^29^ found that the α-Mg dendrite preferred to grow in a direction perpendicular to the $\{20\bar{2}5\}$ plane. Pettersen and *co-workers* ^15,30^ found that the primary growth directions of an α-Mg dendrite were $\langle 11\bar{2}0\rangle$ (with six secondary arms) and $\langle 22\bar{4}5\rangle$ (with three secondary arms). Wang^31^ reported that the α-Mg dendrite grew along $\langle 11\bar{2}0\rangle$ with a six-fold symmetry, and extended along $\langle 0001\rangle$ in 3-D. Böttger and *co-workers* ^9,32^ simulated the α-Mg dendrite growth using a phase field model and proposed two sets of preferred growth directions including $\langle 11\bar{2}0\rangle$ and $\langle 0001\rangle$ by formulating a particular anisotropy function. The simulated dendrite grew faster along $\langle 11\bar{2}0\rangle$ but slower along $\langle 0001\rangle$, exhibiting a plate-like shape in 3-D.

Based on synchrotron X-ray tomography and electron backscattered diffraction (EBSD) techniques, very recently, Yang and *co-workers* ^33^ found that the α-Mg dendrite of most magnesium alloys exhibited an eighteen-primary branches pattern in 3-D, with six along $\langle 11\bar{2}0\rangle$ in the basal plane and twelve others along $\langle 11\bar{2}3\rangle$ in non-basal planes. Casari^34^ found similar results on the preferred growth directions of the α-Mg dendrite *via in-situ* synchrotron X-ray radiography and EBSD techniques. Furthermore, the so-called DOT behavior of the α-Mg dendrite from eighteen-primary branches to twelve-primary branches was also observed in Mg-Zn alloys^35^. Guo *et al*.^36^ reported that the solute concentration greatly affected the microstructural morphology of Mg-Zn alloys, resulting in both dendritic and seaweed type grains.

It is certain that the growth of magnesium alloy dendrite is affected by both the type and quantity of the additional elements. However, these existing investigations are mostly focused on describing the morphology transition of the α-Mg dendrite in a rather qualitative manner, and the underlying mechanism that determines such growth preference and morphological patterns in terms of the lattice structure and crystallographic anisotropy, in particular the exact influence of the solute additions on such transition of dendritic pattern, has not yet been completely understood. Although the growth kinetics of the dendrite are highly dependent on the local solute and energy dissipation at solid/liquid interface, the dendritic orientation selection mechanism is primarily determined by the thermodynamic factor based on the anisotropic surface energy associated with the fundamental lattice structure in light of the crystallographic theory^10,16,18,29,37^.

In this work, the influence of solute additions on the α-Mg dendrite growth behavior was investigated on the basis of crystallographic anisotropy. Alloys including binary Mg-Al, Mg-Ba, Mg-Sn, Mg-Ca, Mg-Y and Mg-Zn alloys with various solute concentrations were employed. The orientation-dependent surface energy was determined *via ab-initio* calculations at equilibrium energy condition based on the density functional theory (DFT) and the *hcp* lattice structure. The growth tendency or orientation selection mechanism of the α-Mg dendrite was analyzed in terms of the crystallographic anisotropy. Results showed that the addition of solute elements could modify the surface energy of magnesium alloys, thus alter the crystallographic anisotropy and the subsequent orientation selection of the α-Mg dendrite. Further analysis revealed that the growth along certain directions of the α-Mg dendrite could be inhibited even a certain magnitude of anisotropy is present.

## Dendritic Morphology and Orientation Selection of Binary Mg-Al/Ba/Sn/Ca/Y Alloys

**Figure 1:**
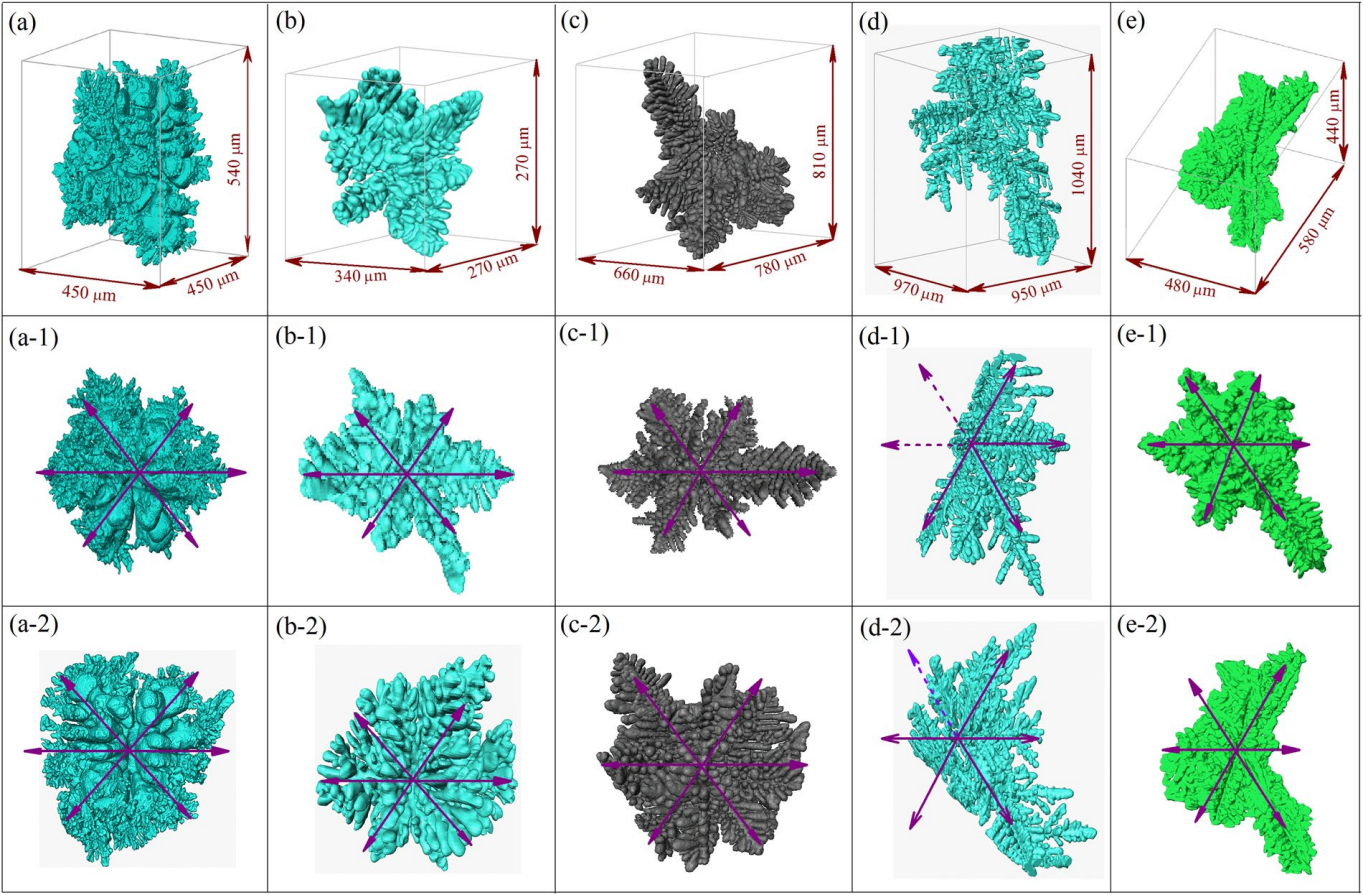
Dendritic morphology of (**a**) Mg-25wt.%Al, (**b**) Mg-10wt.%Ba, (**c**) Mg-30wt.%Sn, (**d**) Mg-15wt.%Ca, and (**e**) Mg-20wt.%Y alloys, reconstructed by 2-D slice images from synchrotron X-ray tomography experiments. (a-1)-(e-1) show the projection of dendritic morphology along $\langle 0001\rangle$ direction, whereas these along $\langle 10\bar{1}0\rangle$ are shown in (a-2)-(e-2), respectively.

**Figure 2:**
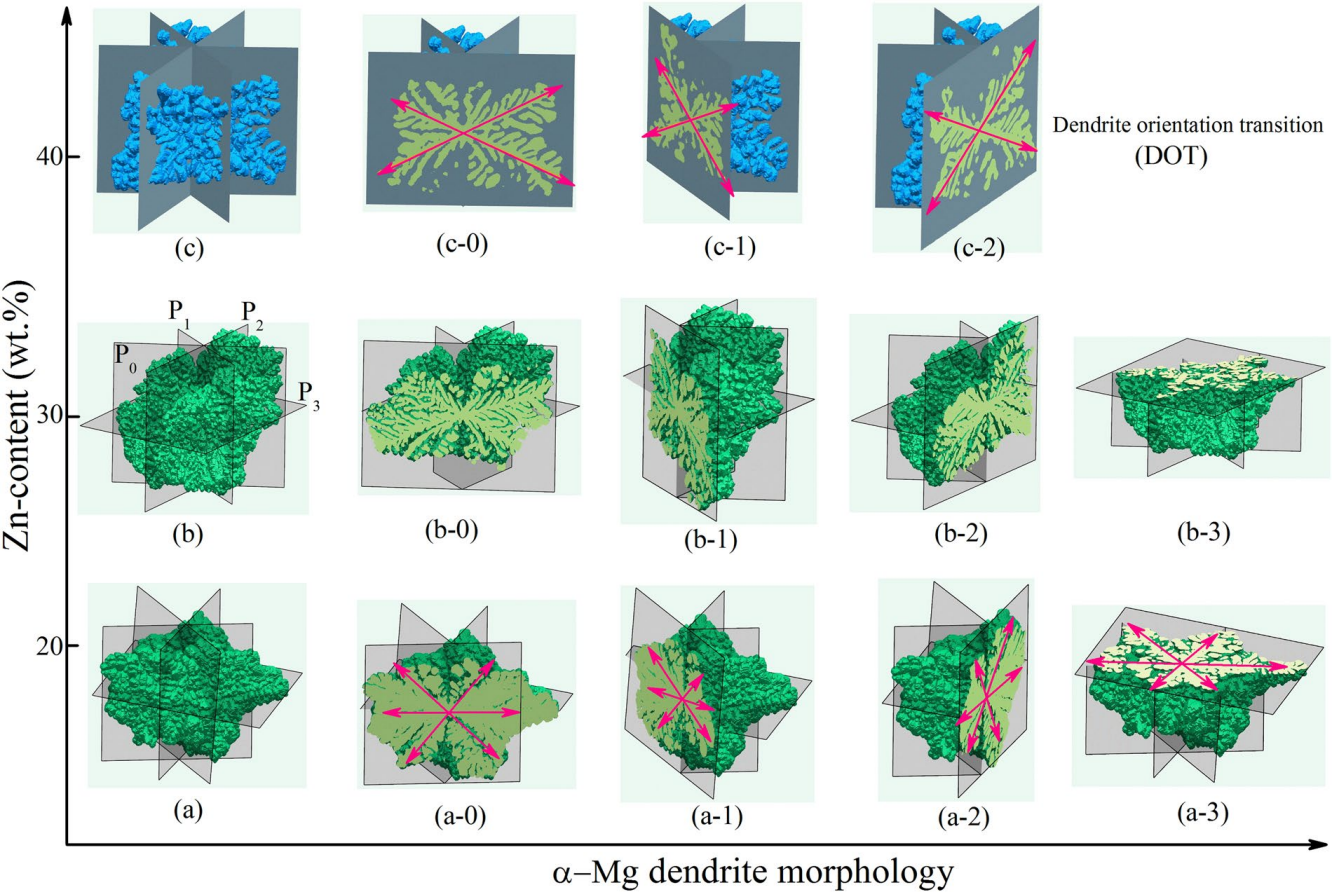
Dendritic morphology of binary Mg-Zn alloys with Zn-contents changing from 20wt.% to 40wt.% with a 10wt.% increment, reconstructed by 2-D slice images from the synchrotron X-ray tomography experiments. (**a**–**c**) show the 3-D dendritic morphology of these Mg-Zn alloys, (a-0)-(c-2) show the corresponding 2-D morphology sections of the α-Mg dendrite by P_0_, P_1_, P_2_ and P_3_ planes, respectively. Those relevant sections used to cut the α-Mg dendrite of these Mg-Zn alloys are illustrated in (**b**), where P_0_, P_1_ and P_2_ are the longitudinal sections with 60° angle away from each other, while P_3_ is the cross-section that perpendicular to the longitudinal sections.

The reconstructed 3-D dendritic morphology of binary Mg-25wt.%Al, Mg-10wt.%Ba, Mg-30wt.%Sn, Mg-15wt.%Ca and Mg-20wt.%Y alloys from the synchrotron X-ray tomography experiments is shown in Fig. 1(a–e), respectively. Because the complete 3-D morphology of the α-Mg dendrite could be reconstructed and rendered, identification of the dendritic growth patterns could be easily performed. Figure 1(a-1–e-1) show the projections of the α-Mg dendritic morphology along $\langle 0001\rangle$, whereas that along $\langle 10\bar{1}0\rangle$ are shown in Fig. 1(a-2–e-2), respectively. The α-Mg dendrite of binary Mg-25wt.%Al, Mg-10wt.%Ba, Mg-30wt.%Sn, Mg-15wt.%Ca and Mg-20wt.%Y alloys, all have six primary branches with six-fold symmetry in the basal plane, and twelve other primary branches in non-basal planes. The angle between the primary branches in the basal and non-basal planes is ~50°, indicating that the growth pattern of α-Mg dendrite in the non-basal plane is not six-fold symmetrical.

The EBSD characterization^33,35^ showed that, for Mg-25wt.%Al, Mg-10wt.%Ba, Mg-30wt.%Sn, Mg-15wt.%Ca and Mg-20wt.%Y alloys, the preferred growth direction of the α-Mg dendrite in the basal plane is $\langle 11\bar{2}0\rangle$, while that in non-basal plane is $\langle 11\bar{2}3\rangle$ (see Supplementary Figure S1), which is similar to that reported by Pettersen^15,30^. However, the α-Mg dendrite of different Mg-based alloys exhibits different morphology features, in particular on the length of the primary branches and morphology of the secondary and high order arms. Based on the reconstructed 3-D dendritic morphology, it was found that for Mg-25wt.%Al alloy, the length ratio between the $\langle 11\bar{2}3\rangle$ and $\langle 11\bar{2}0\rangle$ primary branches is close to one, while that is higher for Mg-10wt.%Ba, Mg-30wt.%Sn, Mg-15wt.%Ca and Mg-20wt.%Y alloys. Such distinction in growth velocity along different preferred directions could be attributed to the change of the surface energy and related crystallographic anisotropy induced by the addition of solute elements, as will be discussed in subsequent sections.

## Dendritic Morphology and Orientation Selection of Binary Mg-Zn Alloys

The DOT phenomenon was commonly observed during the solidification of Al-Zn alloys^22–24^. Our previous work^35^ confirmed that such DOT behavior could also occur in Mg-Zn alloys. Typical metallographic structures of binary Mg-Zn alloys are presented in Supplementary Figure S2, where the primary phase (i.e. the α-Mg dendrite) is in light gray, while the eutectic phase is in dark gray. As the Zn contents increase from 20wt.% to 45wt.%, the amount of the α-Mg dendrite decreases, and more complicated dendritic morphologies exhibit. The snow-flake dendritic patterns could be commonly observed in the Mg-20wt.%Zn alloy, whereas dendrite with only four branches could be observed in the Mg-45wt.%Zn alloy. Besides, the α-Mg dendrite with fragmented morphologies exhibits in the Mg-30wt.%Zn and Mg-40wt.%Zn alloys.

**Figure 3:**
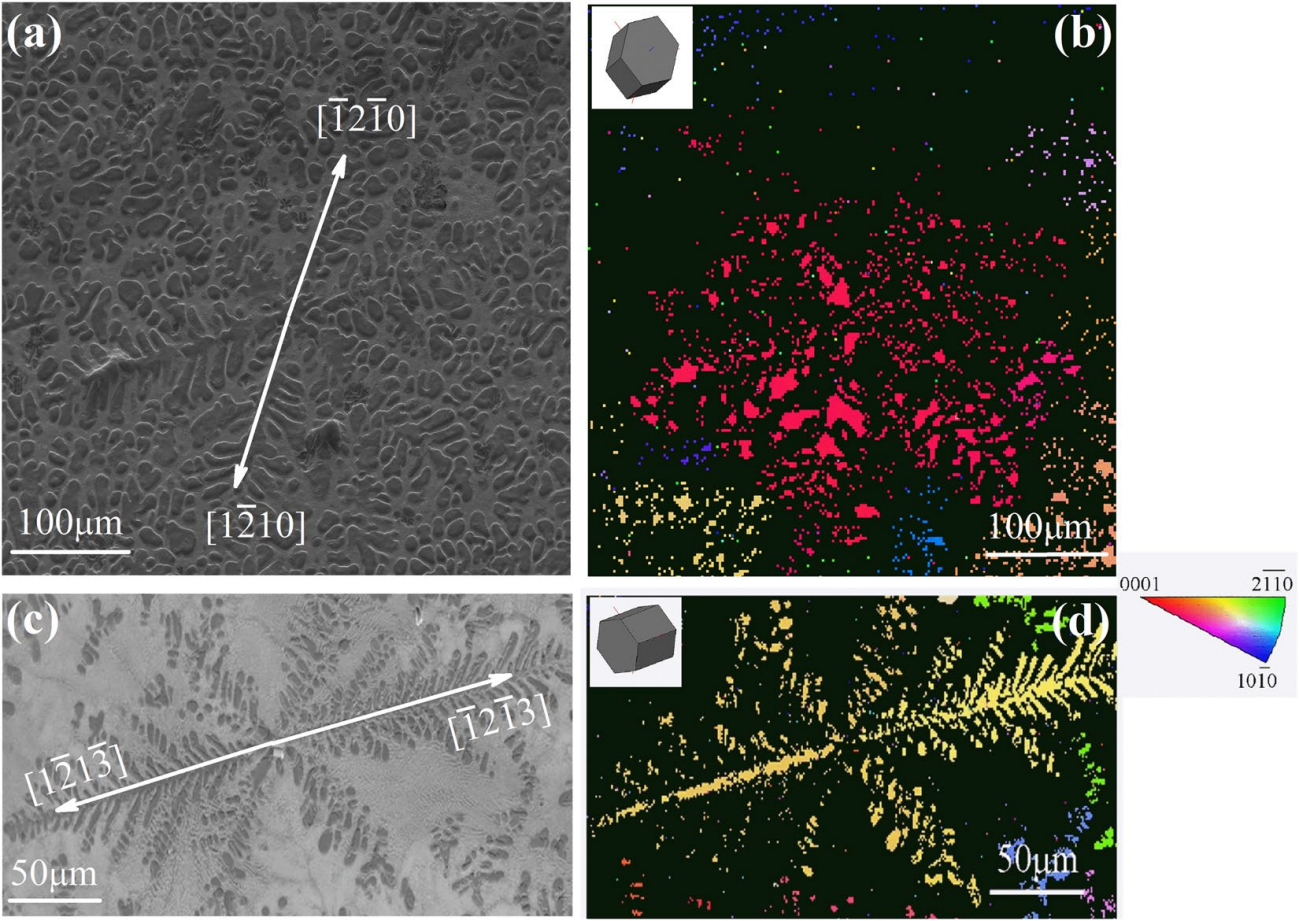
EBSD analysis on the crystallographic orientations of (**a**,**b**) Mg-30wt.%Zn, and (**c**,**d**) Mg-45wt.%Zn alloys, respectively.

The reconstructed 3-D dendritic morphology of the Mg-Zn alloys is shown Fig. 2(a–c). The according growth pattern was further analyzed by cutting the dendrite using specific crystallographic planes. As illustrated in Fig. 2(b), P_0_, P_1_ and P_2_ are the longitudinal sections with a 60° angle away from one another, while P_3_ is the cross-section plane perpendicular to the longitudinal sections. Figure 2(a-0–c-2) show the dendritic patterns of these Mg-Zn alloys on P_0_, P_1_, P_2_ and P_3_ sections, respectively. It was found that the dendritic morphology of binary Mg-20wt.%Zn and Mg-30wt.%Zn alloys is analogous to that of binary Mg-25wt.%Al, Mg-10wt.%Ba, Mg-30wt.%Sn, Mg-15wt.%Ca and Mg-20wt.%Y alloys. With an increase of Zn-contents for Mg-40wt.%Zn alloys, the growth of the α-Mg dendrite branches in the basal plane is retarded, i.e. these branches either became shorter or disappeared. Consequently, the 3-D morphology of the α-Mg dendrite transited from eighteen-primary-branch pattern to twelve-primary-branch pattern. Figure 3 shows the EBSD results on the crystallographic orientations of binary Mg-30wt.%Zn and Mg-45wt.%Zn alloy dendrite. Together with our previous work^35^, it could be concluded that as the Zn-contents increase from 20wt.% to 45wt.%, the preferred growth directions of binary Mg-Zn alloy dendrite transform from $\langle 11\bar{2}0\rangle$ and $\langle 11\bar{2}3\rangle$ to $\langle 11\bar{2}1\rangle$ and $\langle 11\bar{2}3\rangle$, and for the Mg-45wt.%Zn alloy dendrite only growth along $\langle 11\bar{2}3\rangle$ exhibits.

**Figure 4:**
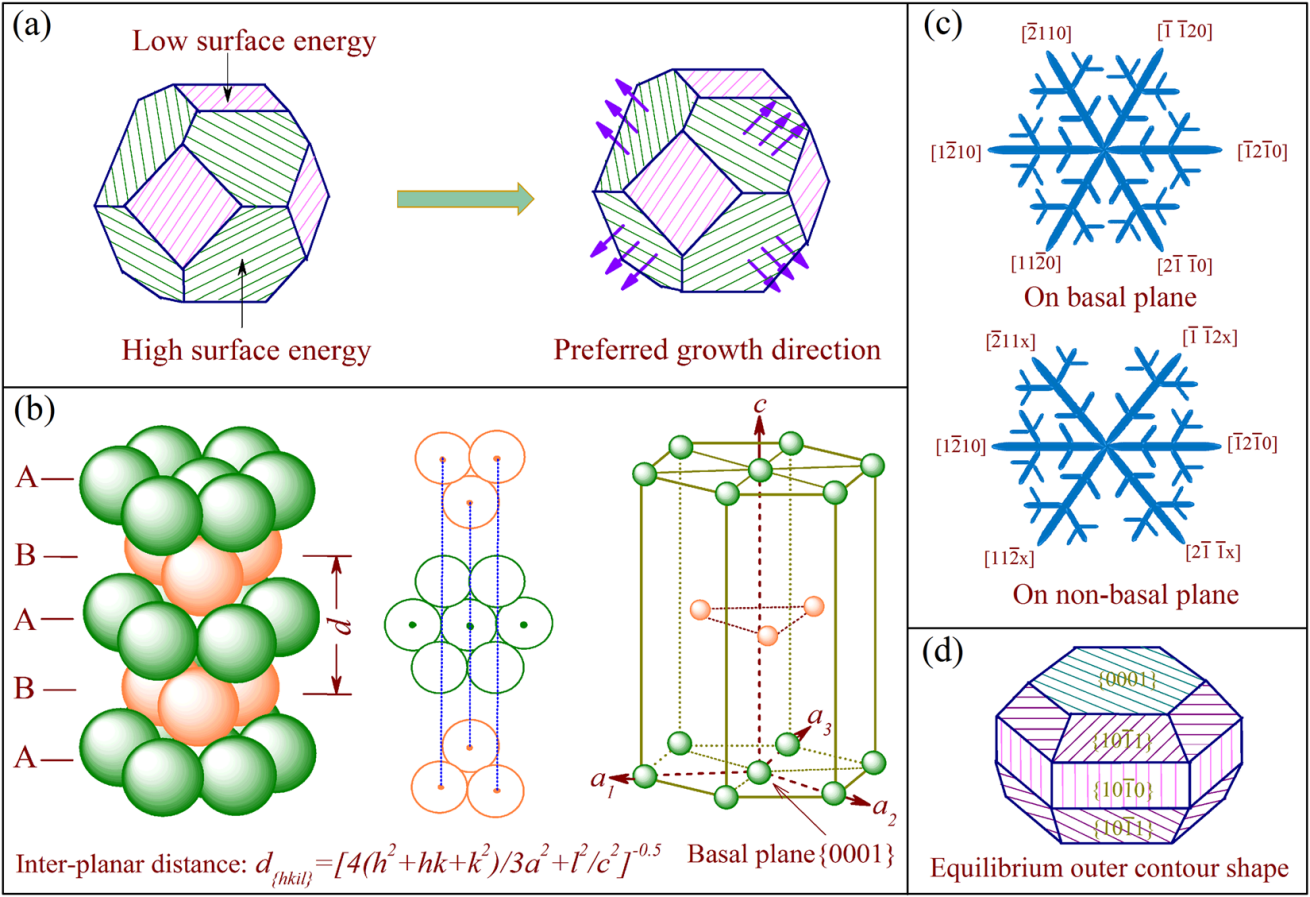
Schematic illustration for the correlation between the surface energy and the growth tendency or orientation selection of dendrite (**a**), the atomic structure of bulk magnesium with hexagonal symmetry (**b**), the ideal growth pattern of the α-Mg dendrite on the basal plane and non-basal planes, respectively (**c**), and the outer contour shape of the α-Mg dendrite at the stable state (**d**).

It has been generally accepted that the dendrite growth tendency or orientation selection is highly dependent on the surface energy and related crystallographic anisotropy^38–43^. The dendrite generally prefers to grow along those directions perpendicular to the crystallographic planes with higher surface energy^44^. As shown in Fig. 4(a), those surface orientations with high surface energy appear as favorable growth directions, and exhibit small facets as dendrite grows^45,46^. Here, we employed and compared the anisotropy of surface energy, which is referred to the $\{0001\}$ basal plane, to understand the dendritic orientation selection of different magnesium alloys^22,47^. Since the dendritic growth tendency or orientation selection is primarily determined by the thermodynamic factor related anisotropic surface energy in light of the underlying lattice structure^40,44,46–48^, in our atomistic model, instead of simulating the real solid/liquid phase transition in relation to growth kinetics during solidification, we assume a solid-vacuum interface and focus on equilibrium energy condition where the anisotropy of solid-liquid interface can be reflected by the solid-vapor interface based on the fundamental *hcp* lattice structure^28,39,49^.

It is worth stressing that the dendritic microstructure could be affected by the kinetic effects, which is highly dependent on the undercooling and local solute redistributions near the solid/liquid interface during rapid solidification. With this respect, our experimental investigation found that for most Mg-based alloys (including Mg-Sn, Mg-Ba, Mg-Y, Mg-Ca etc.), changing the kinetic factors, including quenching temperature, cooling rate and initial concentration of additional elements, would not change the eighteen-primary-branch pattern of the α-Mg dendrite. The difference was only on morphology of the secondary arms like spacing and size. In this respect, the dendritic growth pattern and orientation selection was mostly determined by basic thermodynamic effect rather than kinetic effect.

**Figure 5:**
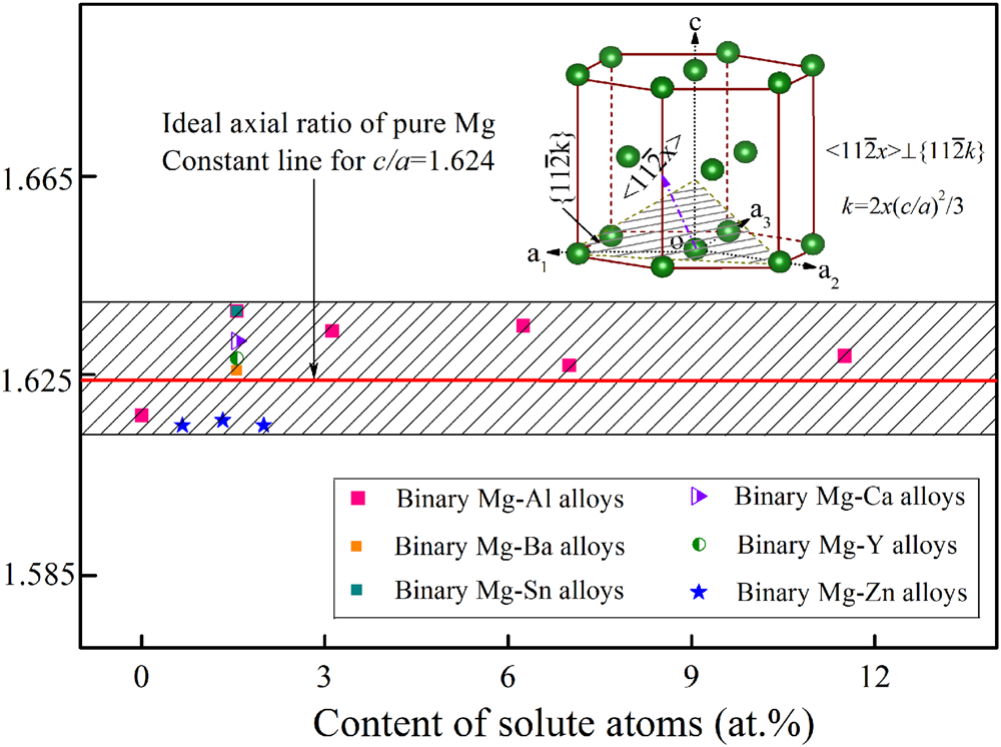
Optimized axial ratio *c/a* of binary dilute Mg-Al/Ba/Sn/Ca/Y/Zn alloys. Insert map illustrates the crystallographic plane $\{11\bar{2}k\}$ and the corresponding orientation $\langle 11\bar{2}x\rangle$ that perpendicular to each other for pure Mg with *hcp* symmetrical structure.

According to our experimental results, those crystallographic planes, including $\{0001\}$, $\{10\bar{1}0\}$, $\{10\bar{1}1\}$, $\{11\bar{2}0\}$, and $\{11\bar{2}k\}$ with *k* ranging from 1 to 8, were considered to investigate the growth behavior of magnesium alloy dendrite. For the *hcp* lattice structure, the basic structural parameters are firmly related to the axial ratio *c/a* ^15,28^. Figure 4(b) shows the schematic illustration for Mg *hcp* structure with ‘ABABAB’ atomic stacking sequence. The crystallographic index of those planes and the according orientations is determined by the *c/a*-ratio, e.g. $k=2x{(c/a)}^{2}/3$ for $\{11\bar{2}k\}$⊥$\langle 11\bar{2}x\rangle$. Accordingly, the $\{11\bar{2}k\}$ plane that perpendicular to the $\langle 11\bar{2}x\rangle$ orientation can be easily obtained based on the optimized *c/a*-ratio, as shown in Fig. 5. For instance, the crystallographic planes perpendicular to $\langle 11\bar{2}0\rangle$ and $\langle 11\bar{2}3\rangle$ are $\{11\bar{2}0\}$ and $\{11\bar{2}5\}$, respectively, while that perpendicular to $\langle 22\bar{4}5\rangle$ is $\{11\bar{2}4\}$. To achieve a quantitative understanding on dendritic orientation selection and growth pattern of magnesium alloys, the surface energy and related crystallographic anisotropy were determined by performing the DFT-based calculations, with attention focused on the influence of both the type and amount of the additional elements.

## Surface Energy and Related Anisotropy of Binary Mg-Al/Ba/Sn/Ca/Y Alloys

**Figure 6:**
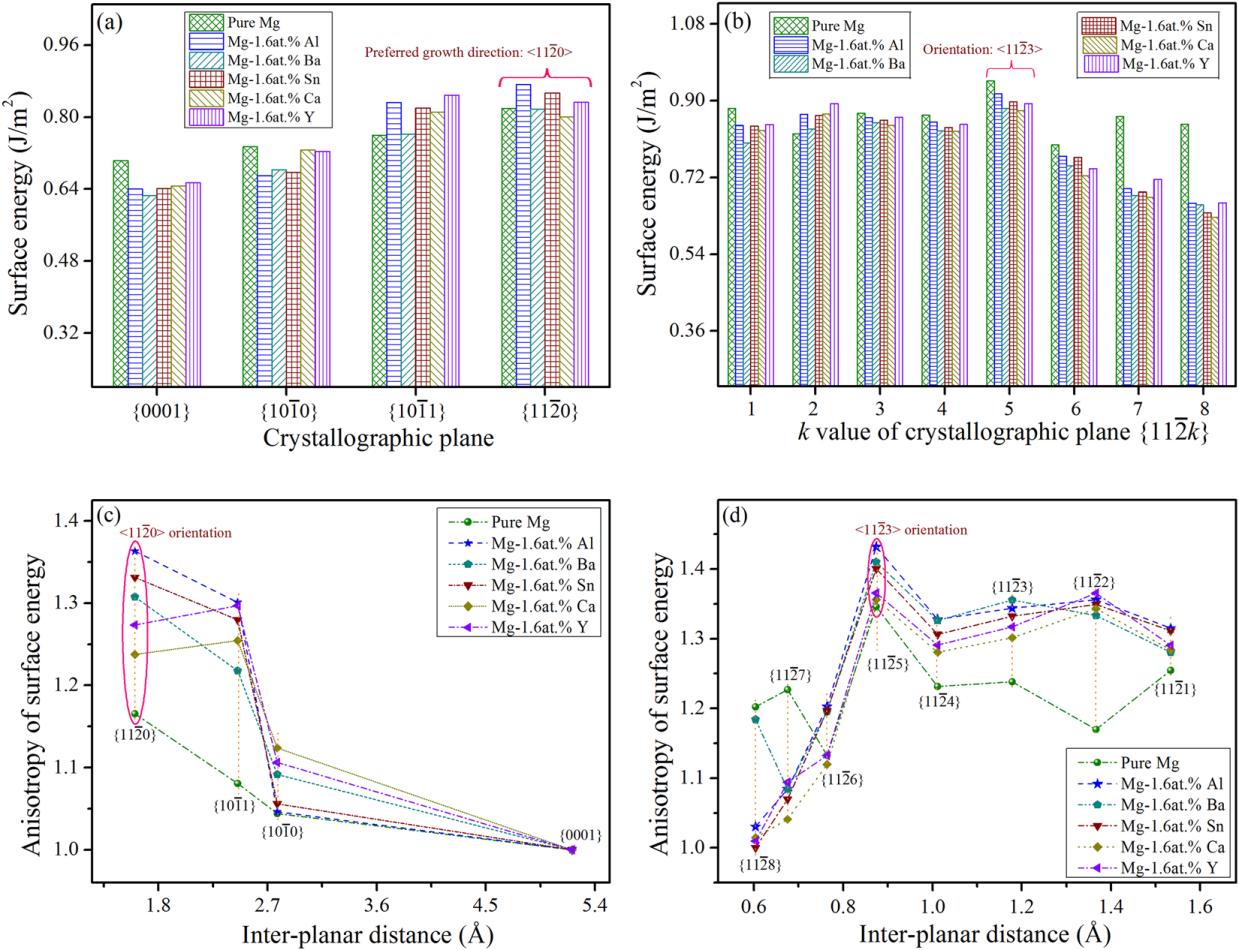
Orientation-dependent surface energy of dilute Mg-Al/Ba/Sn/Ca/Y alloys with a solute concentration of 1.6 at.%. (**a**) shows the surface energy along surface orientations of $\langle 0001\rangle$, $\langle 10\bar{1}0\rangle$, $\langle 10\bar{1}1\rangle$ and $\langle 11\bar{2}0\rangle$, while (**b**) shows that along $\langle 11\bar{2}x\rangle$ surface orientations, (**c**,**d**) show the corresponding anisotropy of surface energy.

Figure 6 shows the determined surface energy along different surface orientations of the binary Mg-Al/Ba/Sn/Ca/Y alloys with 1.6 at.% solute, and the according concrete values for each case are listed in Table 1. Comparing Fig. 6(a) and (b) enables one to find that the surface energy of those high symmetrical planes with low index, e.g. $\{0001\}$, $\{10\bar{1}0\}$ and $\{10\bar{1}1\}$, is slightly lower than that of those high index crystallographic planes like $\{11\bar{2}k\}$. This indicates that those high symmetrical planes are energetically more favorable, and form the final remaining planes with respect to the outer contour of the α-Mg dendrite, as shown in Fig. 4(d). For these dilute Mg-Al/Ba/Sn/Ca/Y alloys, the surface energy of $\langle 11\bar{2}0\rangle$ in the basal plane is higher than that of $\langle 10\bar{1}0\rangle$, whereas in non-basal planes, $\langle 11\bar{2}3\rangle$ has the maximum surface energy than others. Figure 6(c) and (d) show the according anisotropy of surface energy referred to the basal plane, and the corresponding values are listed in Supplementary Table SI. For these Mg-Al/Ba/Sn/Ca/Y alloys, the anisotropy of $\langle 11\bar{2}0\rangle$ in the basal plane is higher than that of $\langle 10\bar{1}0\rangle$, and in non-basal planes, the orientation with the maximum anisotropy of surface energy is $\langle 11\bar{2}3\rangle$. Because the preferred growth directions are those with the high surface energy and related crystallographic anisotropy^44–46^, the calculation results indicate that the α-Mg dendrite of these dilute Mg-Al/Ba/Sn/Ca/Y alloys should prefer to grow along $\langle 11\bar{2}0\rangle$ in the basal plane, and $\langle 11\bar{2}3\rangle$ in non-basal planes. These theoretical predictions for dendritic orientation selection agree well with what we found in experiments on the Mg-Al/Ba/Sn/Ca/Y alloys^33,35^.

**Table 1:** Surface energy (J/m^2^) of pure Mg and binary Mg-Al/Ba/Sn/Ca/Y/Zn alloys with different amount of additional elements, together with other values from literature.

Alloy Composition	Surface energy (J/m^2^)												Reference
	{0001}	$\{\mathrm{10}\bar{{\bf{1}}}0\}$	$\{\mathrm{10}\bar{{\bf{1}}}1\}$	$\{\mathrm{11}\bar{{\bf{2}}}0\}$	$\{\mathrm{11}\bar{{\bf{2}}}1\}$	$\{\mathrm{11}\bar{{\bf{2}}}2\}$	$\{\mathrm{11}\bar{{\bf{2}}}3\}$	$\{\mathrm{11}\bar{{\bf{2}}}4\}$	$\{\mathrm{11}\bar{{\bf{2}}}5\}$	$\{\mathrm{11}\bar{{\bf{2}}}6\}$	$\{\mathrm{11}\bar{{\bf{2}}}7\}$	$\{\mathrm{11}\bar{{\bf{2}}}8\}$	
Pure Mg	0.7030	0.7340	0.7598	0.8193	0.8819	0.8223	0.8705	0.8657	0.9463	0.7963	0.8626	0.8452	This work
	0.7600	—	—	—	—	—	—	—	—	—	—	—	ref.^41^
	0.6410	0.7560	0.8110	—	0.9030	0.8860	0.9110	—	—	—	—	—	ref.^69^
	0.4060	—	—	—	—	—	—	—	—	—	—	—	ref.^42^
Mg-11.5 at.%Al	0.6271	0.7637	0.7677	0.8923	0.8993	0.8517	0.9650	0.9632	0.9367	0.8169	0.7629	0.7453	This work
Mg-7.0 at.%Al	0.6504	0.7295	0.8384	0.8591	0.8898	0.9092	0.8572	0.9248	0.8229	0.7879	0.6951	0.6558	This work
Mg-6.2 at.%Al	0.6544	0.6828	0.8460	0.8728	0.8797	0.8894	0.8815	0.8661	0.9176	0.7894	0.7151	0.6963	This work
Mg-3.1 at.%Al	0.6450	0.6738	0.8291	0.8737	0.8491	0.8788	0.8640	0.8540	0.9193	0.7768	0.7016	0.6878	This work
Mg-1.6 at.%Al	0.6400	0.6696	0.8326	0.8724	0.8417	0.8677	0.8601	0.8498	0.9164	0.7696	0.6940	0.6594	This work
Mg-1.6 at.%Ba	0.6254	0.6826	0.7616	0.8178	0.8008	0.8338	0.8477	0.8293	0.8819	0.7475	0.6779	0.6554	This work
Mg-1.6 at.%Sn	0.6411	0.6769	0.8204	0.8534	0.8407	0.8650	0.8540	0.8374	0.8977	0.7671	0.6858	0.6367	This work
Mg-1.6 at.%Ca	0.6467	0.7268	0.8113	0.8003	0.8305	0.8685	0.8416	0.8281	0.8770	0.7240	0.6732	0.6563	This work
Mg-1.6 at.%Y	0.6540	0.7235	0.8483	0.8328	0.8441	0.8929	0.8613	0.8443	0.8932	0.7407	0.7156	0.6604	This work
Mg-2.0 at.%Zn	0.6394	0.7241	0.7878	0.8655	0.8847	0.9066	0.9114	0.9110	0.8396	0.8460	0.8015	0.8135	This work
Mg-1.3 at.%Zn	0.6392	0.7227	0.7910	0.8603	0.8850	0.9086	0.9176	0.9135	0.8395	0.8440	0.7981	0.8090	This work
Mg-0.7 at.%Zn	0.6391	0.7238	0.7902	0.8556	0.8903	0.9029	0.9122	0.9143	0.8379	0.8425	0.7961	0.8099	This work

**Figure 7:**
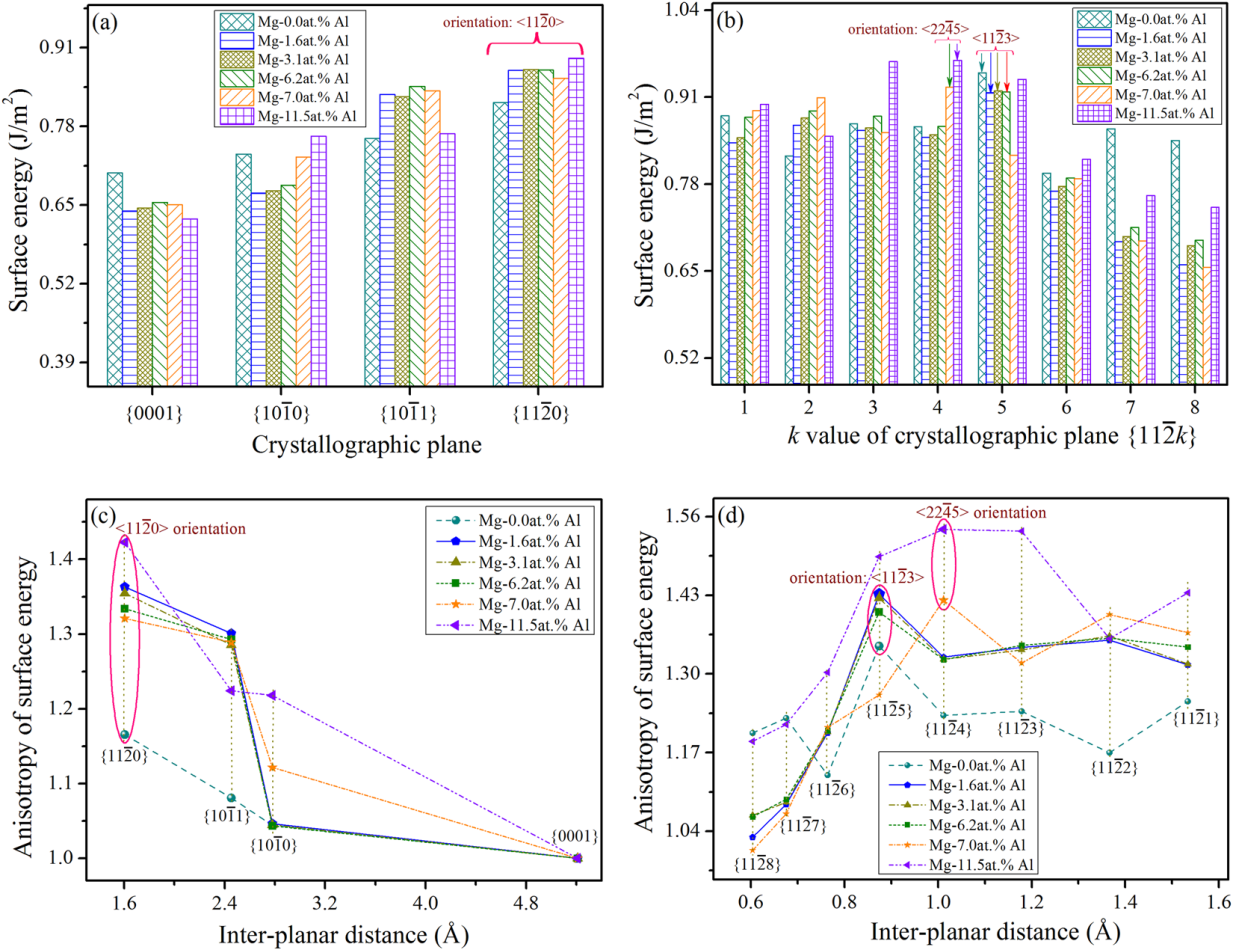
Orientation-dependent surface energy of binary Mg-Al alloys with the Al-contents ranging from 1.6 at.% to 11.5 at.%, and the correlation between anisotropy of surface energy and inter-planar distance. (**a**,**c**) are related to $\{0001\}$, $\{10\bar{1}0\}$, $\{10\bar{1}1\}$ and $\{11\bar{2}0\}$, while (**b**,**d**) are related to $\{11\bar{2}k\}$.

Figure 7(a) and (b) show the determined orientation-dependent surface energy of Mg-Al alloys with compositions of 0.0 at.%Al, 1.6 at.%Al, 3.1 at.%Al, 6.2 at.%Al, 7.0 at.%Al, and 11.5 at.%Al. Because the average composition of solid Mg dendrite is usually lower than the nominal composition of the alloy^5^, the DFT-based calculations were limited to dilute Mg-based alloys, whose compositions were selected based on the solid solubility of Al in matrix Mg, as shown in Table 2. The results indicated that in the basal plane, the surface energy of $\langle 11\bar{2}0\rangle$ is always larger than that of $\langle 10\bar{1}0\rangle$, while in non-basal planes, the maximum surface energy for $\langle 11\bar{2}x\rangle$ changes as the Al-contents increase from 6.2 at.% to 7.0 at.%. Figure 7(c) and (d) show the correlation between inter-planar distance and anisotropy of surface energy, and the concrete values are listed in Supplementary Table SI. For these Mg-Al alloys, the surface energy anisotropy of $\langle 11\bar{2}0\rangle$ in the basal plane is always higher than that of $\langle 10\bar{1}0\rangle$, and in non-basal planes, the surface orientation with the maximum anisotropy of surface energy varies with the magnitude of Al-contents.

**Table 2:** Related information on the additional solute elements of binary Mg-X alloys (X = Al, Ba, Sn, Ca, Y and Zn), including the atomic size (*r*), the solid solubility in magnesium, and the enthalpy of mixing (Δ*H*) between the solute and the solvent.

Elements	*r* (Å)	Maximum Solubility (at.%)	Δ*H* (kJ/mol)
Mg	1.60	—	—
Al	1.43	11.58	−2
Ba	2.17	~0.011	−4
Sn	1.51	3.23	−9
Ca	1.97	0.82	−6
Y	1.80	3.73	−6
Zn	1.39	2.59	−4

## Surface Energy and Related Anisotropy of Binary Mg-Zn Alloys

**Figure 8:**
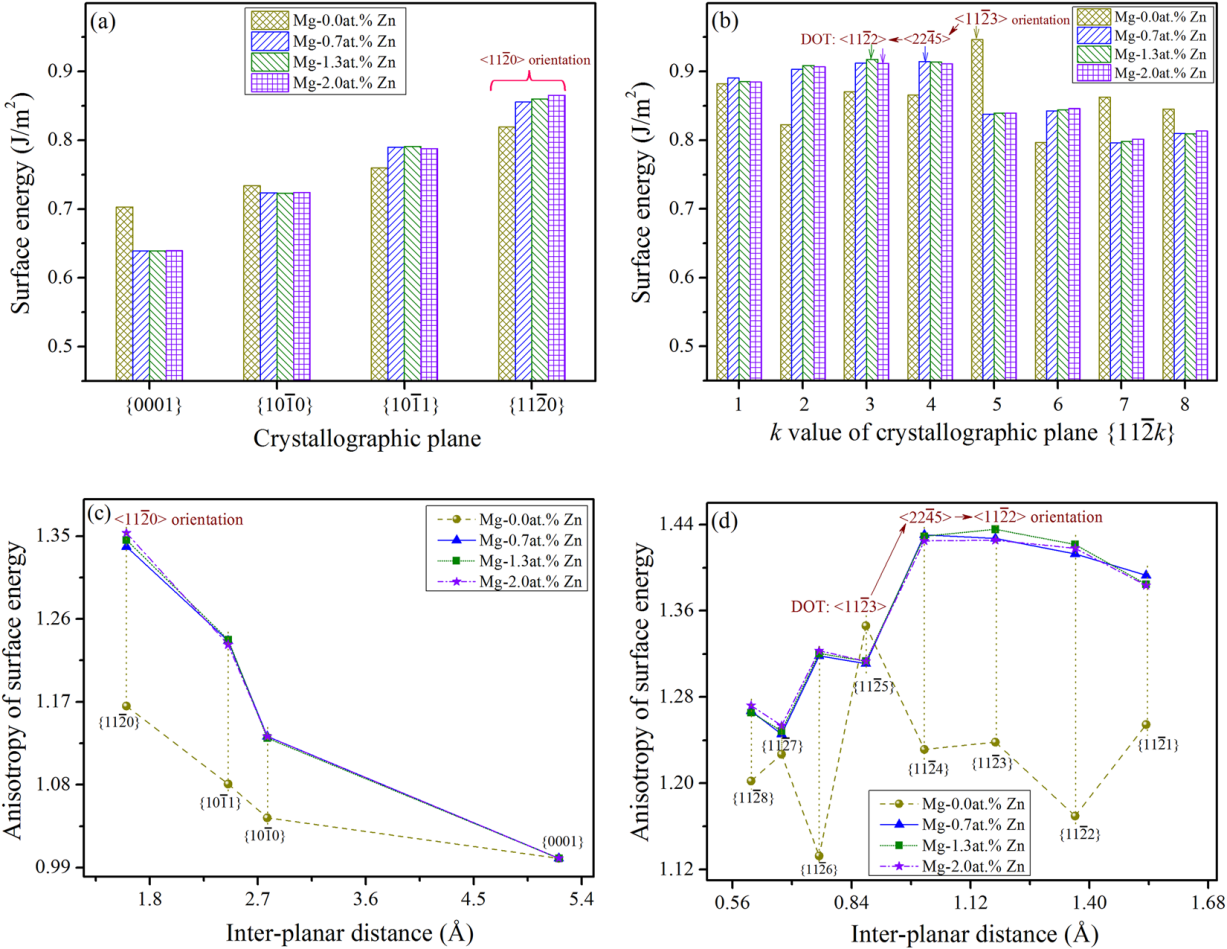
Orientation-dependent surface energy of binary Mg-Zn alloys (**a**,**b**), together with the correlation between anisotropy of surface energy and inter-planar distance (**c**,**d**), indicating that in non-basal planes, the so-called DOT occurred as the Zn-contents varied.

The surface energy of binary Mg-Zn alloys with compositions of 0.7 at.%Zn, 1.3 at.%Zn and 2.0 at.%Zn was determined to study the DOT behavior^34,35^. Similarly, these compositions were chosen based on the solid solubility of Zn in matrix Mg, as shown in Table 2. Figure 8(a) and (b) show the determined orientation-dependent surface energy of these Mg-Zn alloys. As can be seen, the surface energy of $\langle 11\bar{2}0\rangle$ in the basal plane is always higher than that of $\langle 10\bar{1}0\rangle$, and it increases as the Zn-contents increase from 0.7 at.% to 2.0 at.%, whereas in non-basal planes, the maximum surface energy changes as the Zn-contents. Figure 8(c) and (d) show the determined anisotropy of surface energy for these Mg-Zn alloys, and the according concrete values are listed in Supplementary Table SI. In the basal plane, the surface energy anisotropy of $\langle 11\bar{2}0\rangle$ is higher than that of $\langle 10\bar{1}0\rangle$, while in non-basal planes, the highest value of surface energy anisotropy changes from $\langle 11\bar{2}3\rangle$ to $\langle 22\bar{4}5\rangle$ or $\langle 11\bar{2}2\rangle$ as the Zn-contents varied.

Figure 8 indicated that the preferred growth direction of Mg-Zn alloy dendrite in the basal plane is always $\langle 11\bar{2}0\rangle$, whereas that in non-basal planes changes from $\langle 11\bar{2}3\rangle$ to $\langle 11\bar{2}2\rangle$ as Zn-contents increase from 0.0 at.% to 1.3 at.% or 2.0 at.%. For intermediate composition range, as exemplified by Mg-0.7 at.%Zn alloy, the preferred growth direction in non-basal planes becomes $\langle 22\bar{4}5\rangle$. The current *ab-initio* calculations concerning the Mg-Zn alloys agree quite well with these observed from the synchrotron X-ray tomography and EBSD experiments. However, there are still discrepancies between the theoretical predictions and the experimental observations, as will be discussed in subsequent sections.

The dendritic orientation selection and growth pattern of magnesium alloys is dependent on the additional solute elements. As revealed by the *ab-initio* calculations, the presence of the additional elements would cause lattice distortion of the magnesium matrix, degree of which can be evaluated by the *c/a*-ratio (see Fig. 5), based on the atomic size of both solvent and solute, and the atomic interactions reflected by the enthalpy of mixing (Δ*H*)^48,50^. In theory, a large negative Δ*H* value implies a stronger atomic interaction between solvent and solute, and vice versa^51^. As shown in Table 2, in terms of the binary Mg-Al/Ba/Sn/Ca/Y/Zn alloys, the atomic interaction between the solvent and solute elements is different from each other, as well as the atomic size mismatch. Such differences would cause variations in dendritic morphologies and distinctions in dendritic orientation selection of magnesium alloys. Because the growth tendency and morphology transition of the dendrite are extremely sensitive to the surface energy and related crystallographic anisotropy^42,52^, in the following part, the effect of both type and amount of the additional elements on dendritic morphology and growth tendency of magnesium alloys will be discussed.

## Influence of the Additional Elements on the α-Mg Dendrite

Based on the synchrotron X-ray tomography experiments and EBSD characterization, it is clear that the preferred growth directions of the α-Mg dendrite of Mg-25wt.%Al, Mg-10wt.%Ba, Mg-30wt.%Sn, Mg-15wt.%Ca and Mg-20wt.%Y alloys, are $\langle 11\bar{2}0\rangle$ and $\langle 11\bar{2}3\rangle$. The according 3-D dendritic morphology presents an eighteen-primary branch pattern. This is further confirmed *via* the *ab-initio* calculations by the fact that for binary Mg-Al/Ba/Sn/Ca/Y alloys, both $\langle 11\bar{2}0\rangle$ in the basal plane and $\langle 11\bar{2}3\rangle$ in non-basal planes are with higher surface energy than the rest. Furthermore, our prediction that the surface anisotropy of $\langle 11\bar{2}0\rangle$ is higher than $\langle 10\bar{1}0\rangle$ and $\langle 0001\rangle$ *via ab-initio* calculations is in good agreement with that predicted *via* MD simulations^38,52^.

The type of additional elements indeed alters the surface energy and related anisotropy, and thus the dendrite growth tendency along the two preferred growth directions^53^. Figure 6 shows that, comparing with pure magnesium, the surface energy with respect to $\langle 11\bar{2}0\rangle$ increases with the Al, Sn or Y addition, while that is almost unchanged with the addition of Ba or Ca. On the other hand, the surface energy with respect to $\langle 11\bar{2}3\rangle$ decreases by adding Al, Ba, Sn, Ca or Y. The difference of anisotropic surface energy with respect to the preferred growth directions results in variations of the 3-D dendritic morphology. Furthermore, it was found that the influence of Al and Sn on dendritic growth is much more significant than Ba, Ca and Y. This can be understood by comparing the atomic interaction between solvent and solute, as reflected by the Δ*H* value.

Accordingly, the preferred growth direction of the α-Mg dendrite in the basal plane is predicted to be $\langle 11\bar{2}0\rangle$ regardless of the type or concentration of the additional solute, which agrees well with these reported previously^31,32^. The direction in non-basal planes, i.e.$\langle 11\bar{2}3\rangle$, is slightly different from that found by Pettersen and *co-workers* ^15,30^, i.e. $\langle 22\bar{4}5\rangle$. Based on the *ab-initio* calculations, and for the binary Mg-Al alloys, the maximum surface energy in non-basal planes changes from $\langle 11\bar{2}3\rangle$ to $\langle 22\bar{4}5\rangle$ as the Al-contents increased from 6.2 at.% to 7.0 at.%. In this respect, it can be concluded that the preferred growth direction of α-Mg dendrite in the basal plane is always $\langle 11\bar{2}0\rangle$, whereas that in non-basal planes changes from $\langle 11\bar{2}3\rangle$ to $\langle 22\bar{4}5\rangle$ as the Al-contents varied.

## Influence of the Solute Concentration on the α-Mg Dendrite

Both synchrotron X-ray tomography experiment and EBSD characterization showed that for Mg-Zn alloys, the preferred growth directions of the α-Mg dendrite change from a combination of $\langle 11\bar{2}0\rangle$ and $\langle 11\bar{2}3\rangle$ to only $\langle 11\bar{2}3\rangle$ as the Zn-contents increase from 20wt.% to 45wt.%. Accordingly, the 3-D dendritic morphology transform from an eighteen-primary-branch pattern to a twelve-primary-branch pattern. For those Mg-Zn alloys with intermediate Zn-contents, the preferred growth direction became $\langle 11\bar{2}1\rangle$ and a seaweed dendritic morphology exhibited^35,36^.

According to the DFT calculations, as shown in Fig. 8, the preferred growth direction of Mg-Zn alloy dendrite in the basal plane is also $\langle 11\bar{2}0\rangle$ and independent on the amount of solute concentration, i.e. similar as that of other magnesium alloys. In non-basal planes, the preferred growth direction changes from $\langle 11\bar{2}3\rangle$ to $\langle 11\bar{2}2\rangle$ as Zn-contents increase from 0.0 at.% to 1.33 at.% or 2.0 at.%. In particular, for Mg-0.7 at.%Zn alloy dendrite, the preferred growth direction become $\langle 22\bar{4}5\rangle$. Besides, the amount of Zn-contents also alters the dendrite growth tendency along the two preferred growth directions. The anisotropy of surface energy increases as Zn-contents change from 0.7 at.% to 2.0 at.%. According to Fig. 8, the overall 3-D dendritic morphology of these Mg-Zn alloys should present an ideal eighteen-primary-branch pattern, i.e. the α-Mg dendrite exhibits growth tendency along directions within both the basal and non-basal planes, which is different from the experimental result that the Mg-45wt.%Zn alloy dendrite only exhibits the preferred growth direction along $\langle 11\bar{2}3\rangle$ in non-basal planes. This result implies that the growth tendency along certain directions of the α-Mg dendrite could be inhibited even a certain magnitude of anisotropy is present.

Both experiments and *ab-initio* calculations confirmed that the DOT could occur during the dendrite growth of Mg-Zn alloys, i.e. similar to that observed for the Al-Zn alloys^22–24^, signifying that the Zn element indeed has profound influence on the dendritic growth tendency or orientation selection. Besides the underlying crystallographic anisotropy, the DOT behavior of Mg-Zn alloys could be also attributed to the d-electrons of the additional element Zn and the distortion of the lattice structure due to additional element^28,49,54–56^, further investigation is still required to clarify this idea. Based on the *ab-initio* calculations and for binary Mg-Al alloys, the preferred growth direction of the α-Mg dendrite in non-basal planes would change from $\langle 11\bar{2}3\rangle$ to $\langle 22\bar{4}5\rangle$ as the Al-contents increased (see Fig. 7), which agrees quite well with that reported by Pettersen *et al*.^15,30^ because their employed alloy, namely AZ91, contains a rather higher concentration of Al element, i.e. ~9wt.%. However, this alloy also contains certain amount of Zn (0.45~0.9wt.%), and according to our calculations, a minor addition of Zn would alter the preferred growth direction of the α-Mg dendrite from $\langle 11\bar{2}3\rangle$ to $\langle 22\bar{4}5\rangle$ in non-basal planes (see Fig. 8). In this respect, the $\langle 22\bar{4}5\rangle$ preferred growth direction identified by Pettersen *et al*.^15,30^ could be caused by either a high concentration of Al or just an addition of Zn in the magnesium matrix.

It is worth stressing that in the present work, the *ab-initio* calculations on the anisotropic surface energy were performed at the temperature of 0 K based on the solid-vacuum surface slab model under equilibrium conditions, which is different from the practical solid-liquid interface during solidification. Our perspective and focus were to distinguish the variation of surface energy and related crystallographic anisotropy of magnesium alloy dendrite with *hcp* lattice structure, given that the lattice structure is the most important and fundamental factor determining the dendritic growth direction and pattern formation^18,29,37^. Remarkably, even with this temperature difference, the predicted results on the dendritic orientation selection of magnesium alloys were in consistent with the experimental findings. In this respect, it is clear that there exists strong correlation between the interfacial energy variation of anisotropic solid-molten-liquid interface and that of anisotropic solid-vacuum interface. However, the intrinsic reasons for this correlation are still unclear. More investigation, in particular, relevant atomistic simulations at levitated temperature are thus required to further clarify this uncertainty and make clear the exact influence of temperature on dendritic growth behavior.

In conclusion, the orientation selection behavior of binary magnesium alloy dendrite was investigated, with particular attention focused on the influence of both type and amount of the additional elements. Based on both synchrotron X-ray tomography experiment and theoretical *ab-initio* calculations, the underlying mechanism determining the growth tendency or orientation selection of magnesium alloy dendrite was investigated in terms of the surface energy related crystallographic anisotropy based on the *hcp* lattice structure. For these currently studied binary magnesium alloys, including Mg-Al, Mg-Ba, Mg-Sn, Mg-Ca, Mg-Y and Mg-Zn, it was found that the preferred growth direction of the α-Mg dendrite in the basal plane is always $\langle 11\bar{2}0\rangle$ and independent on the additional elements, whereas that in non-basal planes changes with the amount of the additional elements. For Mg-Al alloys, this growth direction changes from $\langle 11\bar{2}3\rangle$ to $\langle 22\bar{4}5\rangle$ as the Al-contents increased. However, for Mg-Zn alloys, this growth direction changes from $\langle 11\bar{2}3\rangle$ to $\langle 22\bar{4}5\rangle$ and/or $\langle 11\bar{2}2\rangle$ as the Zn-contents varied. These theoretical results agree quite well with that found in experiments, and thus confirm that the addition of the Zn element effectively promotes the DOT behavior for both Mg-Zn and Al-Zn alloys.

## Methods

### Synthesis, processing and experimental characterization

The 99.95 wt.% pure magnesium and pure additional elements were used to prepare binary magnesium alloys, including Mg-Al, Mg-Ba, Mg-Sn, Mg-Ca, Mg-Y, and Mg-Zn. The alloys were firstly melted under a mixture of N_2_ and SF_6_ gas atmosphere, then solidified in a permanent mould, and finally quenched in water^8,35^. The samples were then machined into rods of 1.0 mm in diameter and 5.0 mm in height for synchrotron X-ray tomography experiments at the beamline BL13W1 in Shanghai Synchrotron Radiation Facility. A total number of 900 slice images were collected to reconstruct the 3-D dendritic morphology using a software namely Avizo^57^. Cubic specimens of 5 × 5 × 5 mm^3^ processed by chemical etching and electro polishing were prepared for EBSD measurement, which was performed on a TESCAN MIRA3 LMH SEM with HKL Channel 5 system. Dendritic grains with clearly detected primary branches were identified in the metallographic section. The orientation of the α-Mg dendrite was measured *via* EBSD with an angle deviation of ±3° between the dendritic branch and the reference direction. Detail of the apparatus parameters and image processing procedures can be found elsewhere^33^.

### Theoretical calculation scheme

The crystallographic information of magnesium was retrieved from Pearson handbook^58^, and the atomic structure of binary magnesium alloys was constructed using a solid solution model^5,59^, where certain number of solvent atoms were substituted randomly by the selected solute atoms. Accordingly, the slab model^37,60^ was used to simulate the surface atomic structure, and different surface slab models including $\{0001\}$, $\{10\bar{1}0\}$, $\{10\bar{1}1\}$, $\{11\bar{2}0\}$ and $\{11\bar{2}k\}$ with *k* ranging from 1 to 8, were obtained based on our experimental results. Convergence numerical tests with respect to the supercell size, the slab thickness, the vacuum thickness and the number of relaxed atomic layers of the slab model, were performed to ensure the accuracy of the computational scheme. Relevant information on the resultant size of these slab models are provided in Supplementary Table SII.

The *ab-initio* calculations were performed within the framework of density functional theory (DFT), as implemented in the Vienne Ab initio Simulation Package (VASP)^61–63^. The exchange and correlation interaction was described in local density approximation (LDA)^64^. The interaction between ions and valence electrons was modeled by the projector-augmented wave (PAW) potentials^65^. The pseudopotentials employed in this work treated two valence electrons for magnesium (Mg 3 s^2^), three for aluminum (Al 3s^2^3p^1^), ten for barium (Ba 5s^2^5p^6^6s^2^), four for tin (Sn 5s^2^5p^2^), eight for calcium (Ca 3p^6^4s^2^), twelve for zinc (Zn 3d^10^4s^2^) and eight for yttrium (Y 4p^5^4d^1^5s^2^). A plane wave cutoff energy of 420 eV was used for Mg-Ba/Ca/Sn/Y/Al alloys containing 1.6 at.%, 3.1 at.%, 6.2 at.%, 7.0 at.% and 11.5 at.% solute atoms, and 400 eV for Mg-Zn alloys with 0.67 at.%, 1.33 at.% and 2.0 at.% solute atoms, respectively. Brillouin zone integration was modeled by using the Monk-Horst-Pack k-point mesh^66^, and the k-points separation in the Brillouin zone of the reciprocal space was set as 0.01 Å^−1^ for each surface unitcell. The resultant k-point mesh was listed in Supplementary Table SII. The total energy was converged to 5 × 10^−7^ eV/atom with respect to electronic, ionic and unitcell degrees of freedom.

The surface energy is used to analyze the dendritic growth tendency or orientation selection of magnesium alloys. It is defined as the energy required to form a unit area of surface^67–69^. For an *n*-layer slab model, the surface energy can be obtained *via* the following formula:

$${E}_{surf}^{\{hkil\}}=({E}_{n}^{slab}-n{E}_{b})/(2A)$$

where ${E}_{n}^{slab}$ is the total energy of surface slab unitcell, *E*_*b*_ is the total energy of bulk unitcell, *A* is the area of surface slab unitcell, and the factor of 2 denotes the two equivalent surfaces in the particular slab model. The according surface energy was satisfactorily converged to < 0.001 eV/Å^2^ in the present work. The anisotropy of surface energy is referred to the close packed plane of the *hcp* lattice structure, analogous to that of the *fcc* lattice structure^28^, i.e. $\alpha ={E}_{surf}^{\{hkil\}}/{E}_{surf}^{\{0001\}}$. Meanwhile, relevant calculations associated with the position of solute atoms in the supercell were performed to confirm the validity of solid solution model for binary magnesium alloys. Taking binary Mg-Al alloys for instance, it was demonstrated that the calculated results are independent on the position of additional atoms in the solid solution model, as exemplified by the orientation-dependent surface energy for five different cases of Mg-1.6 at.%Al alloys and two different cases of Mg-6.2 at.%Al alloys (see Supplementary Figure S3).

## Electronic supplementary material

Supplementary material
