# Supplementary material for: Correlation between crystallographic anisotropy and dendritic orientation selection of binary magnesium alloys
Source: Sci Rep. 2017 Oct 19;7:13600. doi: 10.1038/s41598-017-12814-5 (PMC5648834; doi:10.1038/s41598-017-12814-5)
Supplement: Supplementary file 1 — Supplementary material [file 41598_2017_12814_MOESM1_ESM.pdf]

Supplementary material for

"Correlation between crystallographic anisotropy and dendritic orientation selection of binary magnesium alloys"

Jinglian Du[1, 2], Zhipeng Guo[1, 2], Ang Zhang[1, 2], Manhong Yang[1, 2], Mei Li[3], Shoumei Xiong[1, 2]

[1]School of Materials Science and Engineering, Tsinghua University, Beijing 100084, China

[2]Laboratory for Advanced Materials Processing Technology, Ministry of Education, Tsinghua University, Beijing 100084, China

[3]Materials Research Department, Research and Innovation Center, Ford Motor Company, MD3182, P.O Box 2053, Dearborn, MI48121, USA

Correspondence and requests for materials should be addressed to Z. G. (zhipeng_guo@mail.tsinghua.edu.cn) and S. X. (smxiong@tsinghua.edu.cn).

This file includes:

- **Computational details of surface energy**
- **Table SI.** Anisotropy of surface energy (i.e. $\alpha = E_{surf}^{\{hkil\}} / E_{surf}^{\{0001\}}$) for pure Mg and binary Mg-Al/Ba/Sn/Ca/Y/Zn alloys with different amount of additional solute elements.
- **Table SII.** Resultant sizes of the atomistic slab model, the angle $\gamma =< o\vec{x}, o\vec{y} >$, and the system size (i.e. atom number) for different surfaces of pure Mg and binary Mg-X (X=Al, Ba, Sn, Ca, Y and Zn) alloys, together with the sampled *k*-point mesh.
- **Figure S1.** Dendritic preferred growth directions of binary Mg-10wt.%Ba alloy, analyzed by the EBSD crystallographic orientation measurements.
- **Figure S2.** Metallographic structure observed from optical microscope for (a) Mg-20wt.%Zn, (b) Mg-30wt.%Zn, (c) Mg-40wt.%Zn, and (d) Mg-45wt.%Zn alloys after water quenching, where the

primary phase (i.e. the α-Mg dendrite) is in light gray, while the eutectic phase is in dark gray.

• **Figure S3.** Orientation-dependent surface energy for five cases of Mg-1.6at.%Al alloy and two cases of Mg-6.2at.%Al alloy with different atomic positions of solvent substituted by solute atoms in magnesium 4×4×2 supercell. (a) and (c) show the high symmetrical surface orientations, (b) and (d) show the high index surface orientations, indicating that in any cases, those crystallographic planes (i.e. $\{11\bar{2}0\}$ and $\{11\bar{2}5\}$) corresponding to the preferred growth direction of magnesium alloy dendrite (i.e. $<11\bar{2}0>$ and $<11\bar{2}3>$) have relatively higher surface energy than those of other surface orientations.

Computational details of surface energy

The surface energy obtained by Eq. (1) usually encounter an unexpected problem of non-convergence caused by the unavoidable discrepancies between the two bulk energies calculated in the bulk form model and the slab form model [*Surf. Sci. 651 (2016) 137.*]. This problem was satisfactorily resolved by substituting the bulk energy E_b in Eq. (1) with the average incremental energy obtained by the subtraction between two slabs with *n* and *n+2* atomic layers (i.e. $E_b = \Delta E(n) = (E_{slab}^{n} - E_{slab}^{n-2})/2$). For each *n* layer, convergence tests were performed with respect to the basis set completeness and the size of the repeating surface unit cell. Particular emphasis was placed on the technical aspects of the calculations, including the convergence tests on the effects of the slab thickness, the vacuum width between slabs and the surface relaxation on surface energy. Furthermore, the LDA pseudopotentials tend to be more accurate than the GGA in surface energy calculation [*J. Phys.: Condens. Mater 18 (2006) L435.*], and accordingly, the PAW-LDA pseudopotentials were used in this work to calculate the anisotropic surface energy of pure Mg and binary Mg-based alloys, including Mg-Al, Mg-Ba, Mg-Sn, Mg-Ca, Mg-Y and Mg-Zn alloys with different solute concentration of additional elements.

Table SI. Anisotropy of surface energy (i.e. $\alpha = E_{surf}^{\{hkil\}} / E_{surf}^{\{0001\}}$) for pure Mg and binary Mg-Al/Ba/Sn/Ca/Y/Zn alloys with different amount of additional solute elements.

Composition		Anisotropy of surface energy											
wt. %	at. %	{0001}	$\{10\bar{1}0\}$	$\{10\bar{1}1\}$	$\{11\bar{2}0\}$	$\{11\bar{2}1\}$	$\{11\bar{2}2\}$	$\{11\bar{2}3\}$	$\{11\bar{2}4\}$	$\{11\bar{2}5\}$	$\{11\bar{2}6\}$	$\{11\bar{2}7\}$	$\{11\bar{2}8\}$
100Mg	100Mg	1	1.0441	1.0807	1.1655	1.2546	1.1697	1.2315	1.2383	1.3461	1.1327	1.2270	1.2022
87.4Mg-12.6Al	88.5Mg-11.5Al	1	1.2179	1.2242	1.4229	1.4314	1.3581	1.5359	1.5389	1.4937	1.3026	1.2166	1.1885
92.3Mg-7.7Al	93.0Mg-7.0Al	1	1.1216	1.2891	1.3210	1.3681	1.3181	1.3980	1.4219	1.2652	1.2115	1.0687	1.0085
93.1Mg-6.9Al	93.7Mg-6.2Al	1	1.1771	1.1943	1.3559	1.3446	1.3399	1.3812	1.4373	1.4634	1.2421	1.1564	1.1421
96.5Mg-3.5Al	96.9Mg-3.1Al	1	1.0448	1.2855	1.3546	1.3165	1.3626	1.3396	1.3241	1.4254	1.2044	1.0877	1.0665
98.3Mg-1.7Al	98.4Mg-1.6Al	1	1.0463	1.3009	1.3632	1.3152	1.3558	1.3438	1.3277	1.4318	1.2026	1.0843	1.0303
91.8Mg-8.2Ba	98.4Mg-1.6Ba	1	1.0916	1.2178	1.3076	1.2804	1.3333	1.3356	1.3261	1.4101	1.1953	1.0840	1.1841
92.8Mg-7.2Sn	98.4Mg-1.6Sn	1	1.0559	1.2797	1.3313	1.3114	1.3493	1.3322	1.3062	1.4004	1.1966	1.0698	1.0002
97.4Mg-2.6Ca	98.4Mg-1.6Ca	1	1.1238	1.2545	1.2376	1.2842	1.3431	1.3014	1.2807	1.3561	1.1196	1.0410	1.0147
94.5Mg-5.5Y	98.4Mg-1.6Y	1	1.1062	1.2970	1.2733	1.2905	1.3653	1.3169	1.2910	1.3656	1.1325	1.0941	1.0097
94.8Mg-5.2Zn	98.0Mg-2.0Zn	1	1.1325	1.2363	1.3386	1.3929	1.4128	1.4272	1.4305	1.3112	1.3183	1.2457	1.2671
96.5Mg-3.5Zn	98.7Mg-1.3Zn	1	1.1307	1.2375	1.3459	1.3846	1.4215	1.4356	1.4292	1.3133	1.3205	1.2486	1.2656
98.2Mg-1.8Zn	99.3Mg-0.7Zn	1	1.1324	1.2363	1.3386	1.3929	1.4127	1.4305	1.4271	1.3111	1.3182	1.2456	1.2671

Table SII. Resultant sizes of the atomistic slab model, the angle $\gamma = \langle o\vec{x}, o\vec{y} \rangle$, and the system size (i.e. atom number) for different surfaces of pure Mg and binary Mg-X (X=Al, Ba, Sn, Ca, Y and Zn) alloys, together with the sampled *k*-point mesh.

Phase	Surface	Atom number	Slab model sizes (Å)			*k*-point mesh	γ (°)
Mg	{0001}	44	*W*=3.209	*L*=3.209	*H*=130.026	15×15×1	120.00
$Mg_{14}Al_2$	-	48	*W*=6.156	*L*=6.143	*H*=42.863	11×11×1	60.066
$Mg_{93}Al_7$	-	300	*W*=15.524	*L*=15.520	*H*=42.870	5×5×1	119.955
$Mg_{60}Al_4$	-	192	*W*=12.413	*L*=12.418	*H*=42.976	5×5×1	120.068
$Mg_{62}Al_2$	-	192	*W*=12.445	*L*=12.449	*H*=43.026	5×5×1	60.096
$Mg_{63}Al$	-	192	*W*=12.468	*L*=12.468	*H*=43.091	5×5×1	119.999
$Mg_{63}Ba$	-	192	*W*=12.614	*L*=12.614	*H*=43.318	5×5×1	119.999
$Mg_{63}Sn$	-	192	*W*=12.492	*L*=12.492	*H*=43.145	5×5×1	119.999
$Mg_{63}Ca$	-	192	*W*=12.547	*L*=12.547	*H*=43.204	5×5×1	119.999
$Mg_{63}Y$	-	192	*W*=12.546	*L*=12.546	*H*=43.118	5×5×1	119.999
$Mg_{147}Zn_3$	-	450	*W*=15.628	*L*=15.628	*H*=57.960	5×5×1	60.014
$Mg_{148}Zn_2$	-	450	*W*=15.647	*L*=15.647	*H*=57.989	5×5×1	60.003
$Mg_{149}Zn$	-	450	*W*=15.665	*L*=15.665	*H*=58.019	5×5×1	60.00
Mg	{$10\bar{1}0$}	44	*W*=3.209	*L*=5.211	*H*=77.294	15×15×1	90.00
$Mg_{14}Al_2$	-	48	*W*=6.143	*L*=10.123	*H*=29.253	9×7×1	89.999
$Mg_{93}Al_7$	-	300	*W*=15.521	*L*=10.106	*H*=53.606	5×5×1	90.048
$Mg_{60}Al_4$	-	192	*W*=12.418	*L*=10.149	*H*=45.448	5×5×1	90.011
$Mg_{62}Al_2$	-	192	*W*=12.448	*L*=10.186	*H*=45.583	5×7×1	89.988
$Mg_{63}Al$	-	192	*W*=12.468	*L*=10.212	*H*=45.602	5×7×1	90.00
$Mg_{63}Ba$	-	192	*W*=12.614	*L*=10.254	*H*=46.102	5×7×1	90.00
$Mg_{63}Sn$	-	192	*W*=12.492	*L*=10.229	*H*=45.658	5×7×1	90.00
$Mg_{63}Ca$	-	192	*W*=12.547	*L*=10.238	*H*=45.860	5×5×1	90.00
$Mg_{63}Y$	-	192	*W*=12.546	*L*=10.215	*H*=45.838	5×5×1	90.00
$Mg_{147}Zn_3$	-	450	*W*=15.631	*L*=15.147	*H*=53.813	5×5×1	89.996
$Mg_{148}Zn_2$	-	450	*W*=15.647	*L*=15.165	*H*=53.850	5×5×1	89.998
$Mg_{149}Zn$	-	450	*W*=15.665	*L*=15.183	*H*=53.896	5×5×1	90.00
Mg	{$10\bar{1}1$}	44	*W*=6.119	*L*=3.209	*H*=69.908	15×15×1	105.202
$Mg_{14}Al_2$	-	48	*W*=11.829	*L*=6.143	*H*=27.246	5×9×1	74.949
$Mg_{93}Al_7$	-	300	*W*=18.512	*L*=15.521	*H*=38.635	5×5×1	114.725
$Mg_{60}Al_4$	-	192	*W*=16.026	*L*=12.418	*H*=35.950	5×5×1	67.236
$Mg_{62}Al_2$	-	192	*W*=16.081	*L*=12.449	*H*=36.025	5×5×1	67.312
$Mg_{63}Al$	-	192	*W*=16.117	*L*=12.468	*H*=36.040	5×5×1	112.756
$Mg_{63}Ba$	-	192	*W*=16.256	*L*=12.614	*H*=36.180	5×5×1	112.828
$Mg_{63}Sn$	-	192	*W*=16.146	*L*=12.492	*H*=36.071	5×5×1	112.758
$Mg_{63}Ca$	-	192	*W*=16.195	*L*=12.547	*H*=36.116	5×5×1	112.792
$Mg_{63}Y$	-	192	*W*=16.179	*L*=12.546	*H*=36.093	5×5×1	112.813
$Mg_{147}Zn_3$	-	450	*W*=21.765	*L*=15.631	*H*=45.005	3×5×1	68.959
$Mg_{148}Zn_2$	-	450	*W*=21.790	*L*=15.647	*H*=45.049	3×5×1	68.959
$Mg_{149}Zn$	-	450	*W*=21.816	*L*=15.665	*H*=45.047	3×5×1	68.958

Table SII (continued)

Phase	Surface	Atomic number	Slab model sizes (Å)			k-point mesh	γ (º)
Mg	$\{11\bar{2}0\}$	44	W=5.211	L=5.559	H=51.699	15×15×1	90.00
$Mg_{14}Al_2$	-	48	W=10.123	L=10.648	H=22.727	5×5×1	89.880
$Mg_{93}Al_7$	-	300	W=10.106	L=26.879	H=36.879	5×3×1	89.928
$Mg_{60}Al_4$	-	192	W=10.149	L=21.512	H=32.132	5×3×1	89.989
$Mg_{62}Al_2$	-	192	W=10.186	L=21.583	H=32.171	5×3×1	90.007
$Mg_{63}Al$	-	192	W=10.212	L=21.596	H=32.171	5×3×1	90.00
$Mg_{63}Ba$	-	192	W=10.254	L=21.848	H=32.379	5×3×1	90.00
$Mg_{63}Sn$	-	192	W=10.229	L=21.637	H=32.183	5×3×1	90.00
$Mg_{63}Ca$	-	192	W=10.238	L=21.733	H=32.272	5×5×1	90.00
$Mg_{63}Y$	-	192	W=10.215	L=21.731	H=32.272	5×5×1	90.00
$Mg_{147}Zn_3$	-	450	W=15.147	L=27.073	H=36.992	5×3×1	90.007
$Mg_{148}Zn_2$	-	450	W=15.165	L=27.102	H=37.003	5×3×1	90.001
$Mg_{149}Zn$	-	450	W=15.183	L=27.133	H=36.972	5×3×1	90.000
Mg	$\{11\bar{2}1\}$	44	W=5.559	L=6.119	H=50.973	9×9×1	117.012
$Mg_{14}Al_2$	-	48	W=10.648	L=11.829	H=23.127	7×5×1	63.326
$Mg_{93}Al_7$	-	300	W=18.512	L=18.528	H=33.230	3×5×1	93.052
$Mg_{60}Al_4$	-	192	W=16.033	L=16.040	H=29.614	5×5×1	84.247
$Mg_{62}Al_2$	-	192	W=16.098	L=16.083	H=29.656	5×5×1	84.237
$Mg_{63}Al$	-	192	W=16.117	L=16.117	H=29.674	5×5×1	84.131
$Mg_{63}Ba$	-	192	W=16.256	L=16.256	H=30.010	5×5×1	84.443
$Mg_{63}Sn$	-	192	W=16.146	L=16.146	H=29.679	5×5×1	84.139
$Mg_{63}Ca$	-	192	W=16.195	L=16.195	H=29.817	5×5×1	84.287
$Mg_{63}Y$	-	192	W=16.179	L=16.179	H=29.785	5×5×1	84.377
$Mg_{147}Zn_3$	-	450	W=21.766	L=21.766	H=35.639	3×3×1	76.913
$Mg_{148}Zn_2$	-	450	W=21.790	L=21.790	H=35.642	3×3×1	76.907
$Mg_{149}Zn$	-	450	W=21.816	L=21.816	H=35.660	3×3×1	76.906
Mg	$\{11\bar{2}2\}$	44	W=5.559	L=6.119	H=46.692	9×9×1	90.00
$Mg_{14}Al_2$	-	48	W=10.648	L=11.829	H=90.033	5×5×1	90.033
$Mg_{93}Al_7$	-	300	W=26.879	L=18.527	H=27.699	3×3×1	90.028
$Mg_{60}Al_4$	-	192	W=21.512	L=16.026	H=25.880	3×5×1	90.029
$Mg_{62}Al_2$	-	192	W=21.583	L=16.081	H=25.863	3×5×1	89.928
$Mg_{63}Al$	-	192	W=21.596	L=16.117	H=25.889	3×5×1	90.00
$Mg_{63}Ba$	-	192	W=21.848	L=16.256	H=26.156	3×5×1	90.00
$Mg_{63}Sn$	-	192	W=21.637	L=16.146	H=25.903	3×5×1	90.00
$Mg_{63}Ca$	-	192	W=21.733	L=16.195	H=26.002	5×5×1	90.00
$Mg_{63}Y$	-	192	W=21.731	L=16.179	H=25.973	5×5×1	90.00
$Mg_{147}Zn_3$	-	450	W=27.073	L=21.765	H=30.993	3×3×1	90.003
$Mg_{148}Zn_2$	-	450	W=27.102	L=21.790	H=31.008	3×3×1	90.001
$Mg_{149}Zn$	-	450	W=27.133	L=21.816	H=31.043	3×3×1	90.00

Table SII (continued)

Phase	Surface	Atomic number	Slab model sizes (Å)			*k*-point mesh	γ (°)
Mg	{11$\bar{2}$3}	44	*W*=5.559	*L*=7.619	*H*=43.341	9×9×1	111.395
$Mg_{14}Al_2$	-	48	*W*=10.648	*L*=14.677	*H*=21.301	7×5×1	111.268
$Mg_{93}Al_7$	-	300	*W*=26.879	*L*=28.726	*H*=24.141	3×3×1	62.121
$Mg_{60}Al_4$	-	192	*W*=21.512	*L*=23.761	*H*=23.238	3×3×1	63.116
$Mg_{62}Al_2$	-	192	*W*=21.583	*L*=23.833	*H*=23.260	3×3×1	116.843
$Mg_{63}Al$	-	192	*W*=21.596	*L*=23.889	*H*=23.263	3×3×1	116.872
$Mg_{63}Ba$	-	192	*W*=21.848	*L*=24.135	*H*=23.518	3×3×1	116.912
$Mg_{63}Sn$	-	192	*W*=21.637	*L*=23.934	*H*=23.255	3×3×1	116.873
$Mg_{63}Ca$	-	192	*W*=21.733	*L*=24.024	*H*=23.348	5×5×1	116.893
$Mg_{63}Y$	-	192	*W*=21.731	*L*=24.013	*H*=23.306	5×5×1	116.904
$Mg_{147}Zn_3$	-	450	*W*=27.073	*L*=31.018	*H*=27.388	3×3×1	64.132
$Mg_{148}Zn_2$	-	450	*W*=27.102	*L*=31.056	*H*=27.413	3×3×1	64.130
$Mg_{149}Zn$	-	450	*W*=27.133	*L*=31.092	*H*=27.357	3×3×1	115.870
Mg	{11$\bar{2}$4}	44	*W*=5.559	*L*=8.267	*H*=39.238	9×9×1	90.00
$Mg_{14}Al_2$	-	48	*W*=10.648	*L*=15.911	*H*=19.954	5×5×1	89.973
$Mg_{93}Al_7$	-	300	*W*=26.879	*L*=32.663	*H*=21.796	3×3×1	90.009
$Mg_{60}Al_4$	-	192	*W*=21.512	*L*=26.802	*H*=22.034	3×3×1	90.031
$Mg_{62}Al_2$	-	192	*W*=21.583	*L*=26.893	*H*=22.057	3×3×1	89.917
$Mg_{63}Al$	-	192	*W*=21.596	*L*=26.947	*H*=21.529	3×3×1	90.00
$Mg_{63}Ba$	-	192	*W*=21.848	*L*=27.232	*H*=22.114	3×3×1	90.00
$Mg_{63}Sn$	-	192	*W*=21.637	*L*=26.998	*H*=21.529	3×3×1	90.00
$Mg_{63}Ca$	-	192	*W*=21.733	*L*=27.103	*H*=21.606	5×5×1	90.00
$Mg_{63}Y$	-	192	*W*=21.731	*L*=27.092	*H*=22.085	5×5×1	90.00
$Mg_{147}Zn_3$	-	450	*W*=27.073	*L*=34.733	*H*=25.207	3×3×1	90.008
$Mg_{148}Zn_2$	-	450	*W*=27.102	*L*=34.775	*H*=25.216	3×3×1	90.002
$Mg_{149}Zn$	-	450	*W*=27.133	*L*=34.816	*H*=25.060	3×3×1	90.00
Mg	{11$\bar{2}$5}	44	*W*=5.559	*L*=9.962	*H*=36.791	9×9×1	106.20
$Mg_{14}Al_2$	-	48	*W*=10.648	*L*=19.142	*H*=19.712	5×3×1	106.103
$Mg_{93}Al_7$	-	300	*W*=26.879	*L*=42.311	*H*=20.848	3×3×1	71.485
$Mg_{60}Al_4$	-	192	*W*=21.512	*L*=34.348	*H*=18.443	3×3×1	71.782
$Mg_{62}Al_2$	-	192	*W*=21.583	*L*=34.454	*H*=20.370	3×3×1	108.168
$Mg_{63}Al$	-	192	*W*=21.596	*L*=34.533	*H*=20.383	3×3×1	108.221
$Mg_{63}Ba$	-	192	*W*=21.848	*L*=34.913	*H*=108.233	3×3×1	108.233
$Mg_{63}Sn$	-	192	*W*=21.637	*L*=34.598	*H*=20.367	3×3×1	108.221
$Mg_{63}Ca$	-	192	*W*=21.733	*L*=34.741	*H*=20.407	5×5×1	108.227
$Mg_{63}Y$	-	192	*W*=21.731	*L*=34.731	*H*=20.390	5×5×1	108.231
$Mg_{147}Zn_3$	-	450	*W*=27.073	*L*=44.034	*H*=23.448	3×3×1	72.106
$Mg_{148}Zn_2$	-	450	*W*=27.102	*L*=44.088	*H*=23.405	3×3×1	72.102
$Mg_{149}Zn$	-	450	*W*=27.133	*L*=44.140	*H*=23.413	3×3×1	107.90

Table SII (continued)

Phase	Surface	Atomic number	Slab model sizes (Å)			k-point mesh	γ (°)
Mg	$\{11\bar{2}6\}$	44	W=5.559	L=10.947	H=34.038	9×9×1	90.00
$Mg_{14}Al_2$	-	48	W=10.648	L=21.029	H=18.769	5×3×1	90.00
$Mg_{93}Al_7$	-	300	W=26.879	L=47.677	H=19.830	3×3×1	90.002
$Mg_{60}Al_4$	-	192	W=21.512	L=38.568	H=19.566	3×3×1	90.03
$Mg_{62}Al_2$	-	192	W=21.583	L=38.699	H=19.562	3×3×1	89.915
$Mg_{63}Al$	-	192	W=21.596	L=38.775	H=19.543	3×3×1	90.00
$Mg_{63}Ba$	-	192	W=21.848	L=39.207	H=19.730	3×3×1	90.00
$Mg_{63}Sn$	-	192	W=21.637	L=38.848	H=19.544	3×3×1	90.00
$Mg_{63}Ca$	-	192	W=21.733	L=39.010	H=19.612	5×5×1	90.00
$Mg_{63}Y$	-	192	W=21.731	L=39.001	H=19.584	5×5×1	90.00
$Mg_{147}Zn_3$	-	450	W=27.073	L=49.271	H=21.831	3×3×1	90.009
$Mg_{148}Zn_2$	-	450	W=27.102	L=49.329	H=21.852	3×3×1	90.002
$Mg_{149}Zn$	-	450	W=27.133	L=49.388	H=21.856	3×3×1	90.00
Mg	$\{11\bar{2}7\}$	44	W=5.559	L=12.691	H=32.518	9×9×1	102.651
$Mg_{14}Al_2$	-	48	W=10.648	L=24.356	H=18.670	5×3×1	102.557
$Mg_{93}Al_7$	-	300	W=26.879	L=56.901	H=19.203	3×1×1	103.662
$Mg_{60}Al_4$	-	192	W=21.512	L=45.854	H=17.550	3×3×1	76.465
$Mg_{62}Al_2$	-	192	W=21.583	L=45.999	H=18.956	3×3×1	103.482
$Mg_{63}Al$	-	192	W=21.596	L=46.102	H=18.955	3×3×1	103.546
$Mg_{63}Ba$	-	192	W=21.848	L=46.623	H=19.221	3×3×1	103.551
$Mg_{63}Sn$	-	192	W=21.637	L=46.188	H=18.934	3×3×1	103.546
$Mg_{63}Ca$	-	192	W=21.733	L=46.385	H=18.976	5×5×1	103.548
$Mg_{63}Y$	-	192	W=21.731	L=46.376	H=18.955	5×5×1	103.550
$Mg_{147}Zn_3$	-	450	W=27.073	L=58.346	H=21.208	3×1×1	76.595
$Mg_{148}Zn_2$	-	450	W=27.102	L=58.418	H=21.213	3×1×1	76.589
$Mg_{149}Zn$	-	450	W=27.133	L=58.487	H=21.211	3×1×1	103.412
Mg	$\{11\bar{2}8\}$	44	W=5.559	L=13.855	H=30.673	9×9×1	90.00
$Mg_{14}Al_2$	-	48	W=10.648	L=26.590	H=18.496	5×3×1	89.922
$Mg_{93}Al_7$	-	300	W=26.879	L=62.943	H=18.599	3×1×1	89.998
$Mg_{60}Al_4$	-	192	W=21.512	L=50.639	H=18.723	3×3×1	90.031
$Mg_{62}Al_2$	-	192	W=21.583	L=50.811	H=18.740	3×3×1	89.913
$Mg_{63}Al$	-	192	W=21.596	L=50.909	H=18.749	3×3×1	90.00
$Mg_{63}Ba$	-	192	W=21.848	L=51.488	H=18.766	3×3×1	90.00
$Mg_{63}Sn$	-	192	W=21.637	L=51.005	H=18.750	3×3×1	90.00
$Mg_{63}Ca$	-	192	W=21.733	L=51.224	H=18.751	5×5×1	90.00
$Mg_{63}Y$	-	192	W=21.731	L=51.215	H=18.753	5×5×1	90.00
$Mg_{147}Zn_3$	-	450	W=27.073	L=64.321	H=20.496	3×1×1	90.011
$Mg_{148}Zn_2$	-	450	W=27.102	L=64.398	H=20.483	3×1×1	90.002
$Mg_{149}Zn$	-	450	W=27.133	L=64.475	H=20.430	3×1×1	90.00

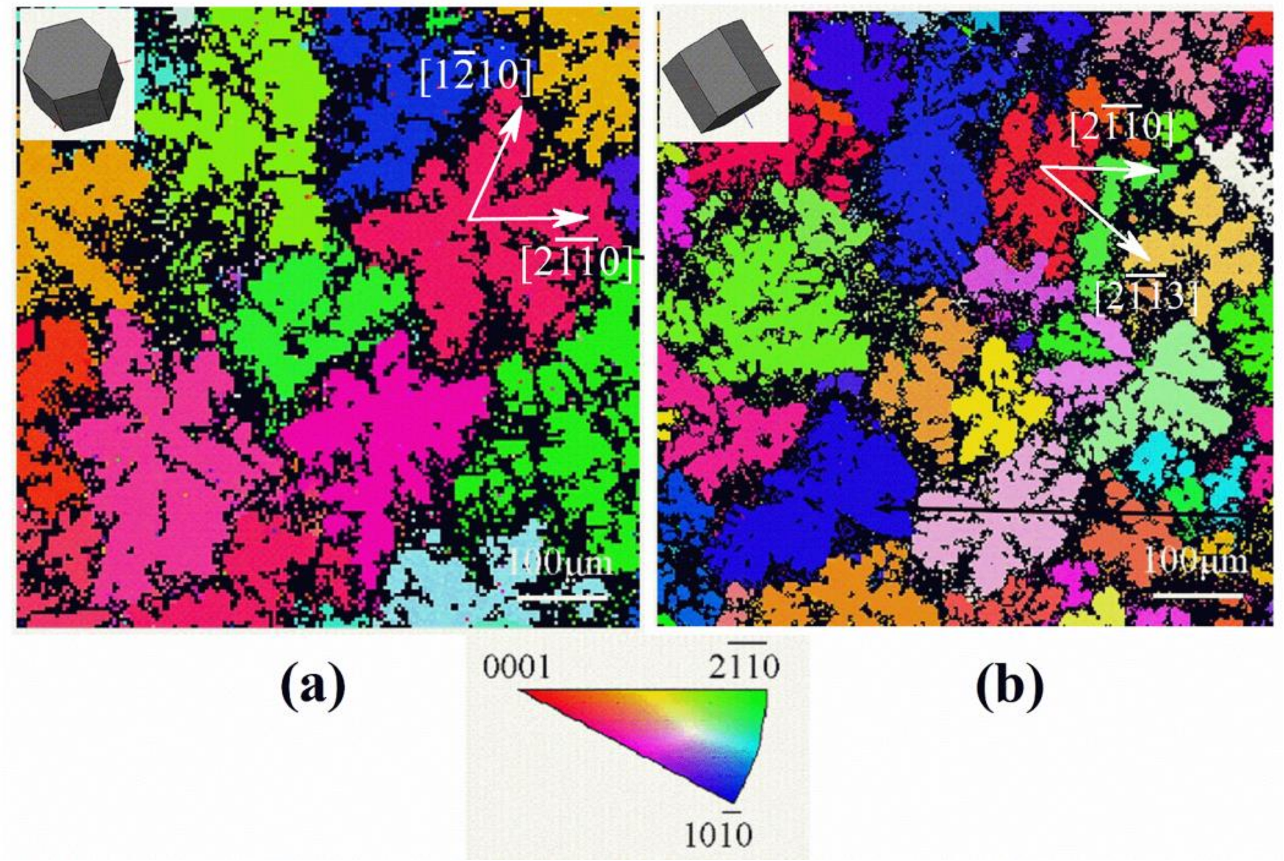

Figure S1. Dendritic preferred growth directions of binary Mg-10wt.%Ba alloy, analyzed by the EBSD crystallographic orientation measurements.

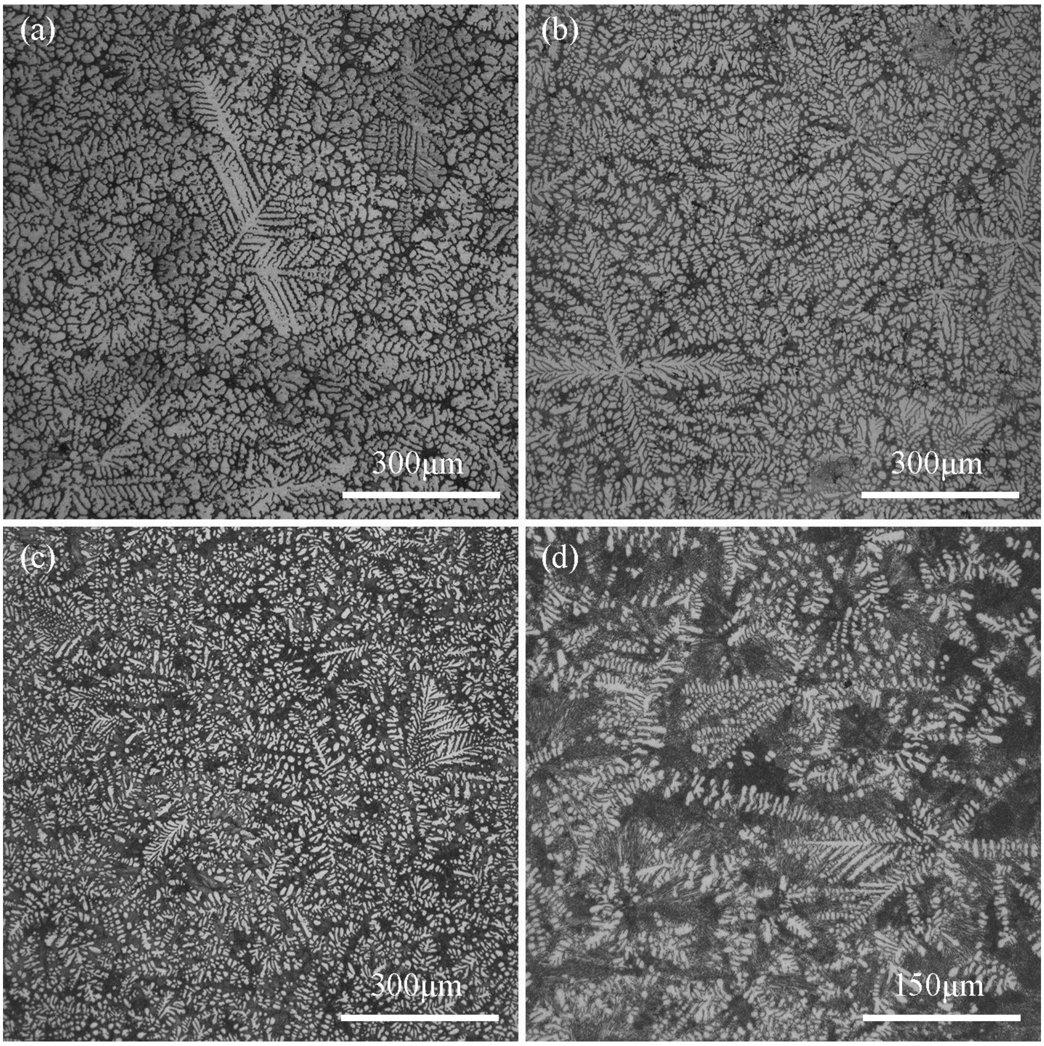

Figure S2. Metallographic structure observed from optical microscope for (a) Mg-20wt.%Zn, (b) Mg-30wt.%Zn, (c) Mg-40wt.%Zn, and (d) Mg-45wt.%Zn alloys after water quenching, where the primary phase (i.e. the α-Mg dendrite) is in light gray, while the eutectic phase is in dark gray.

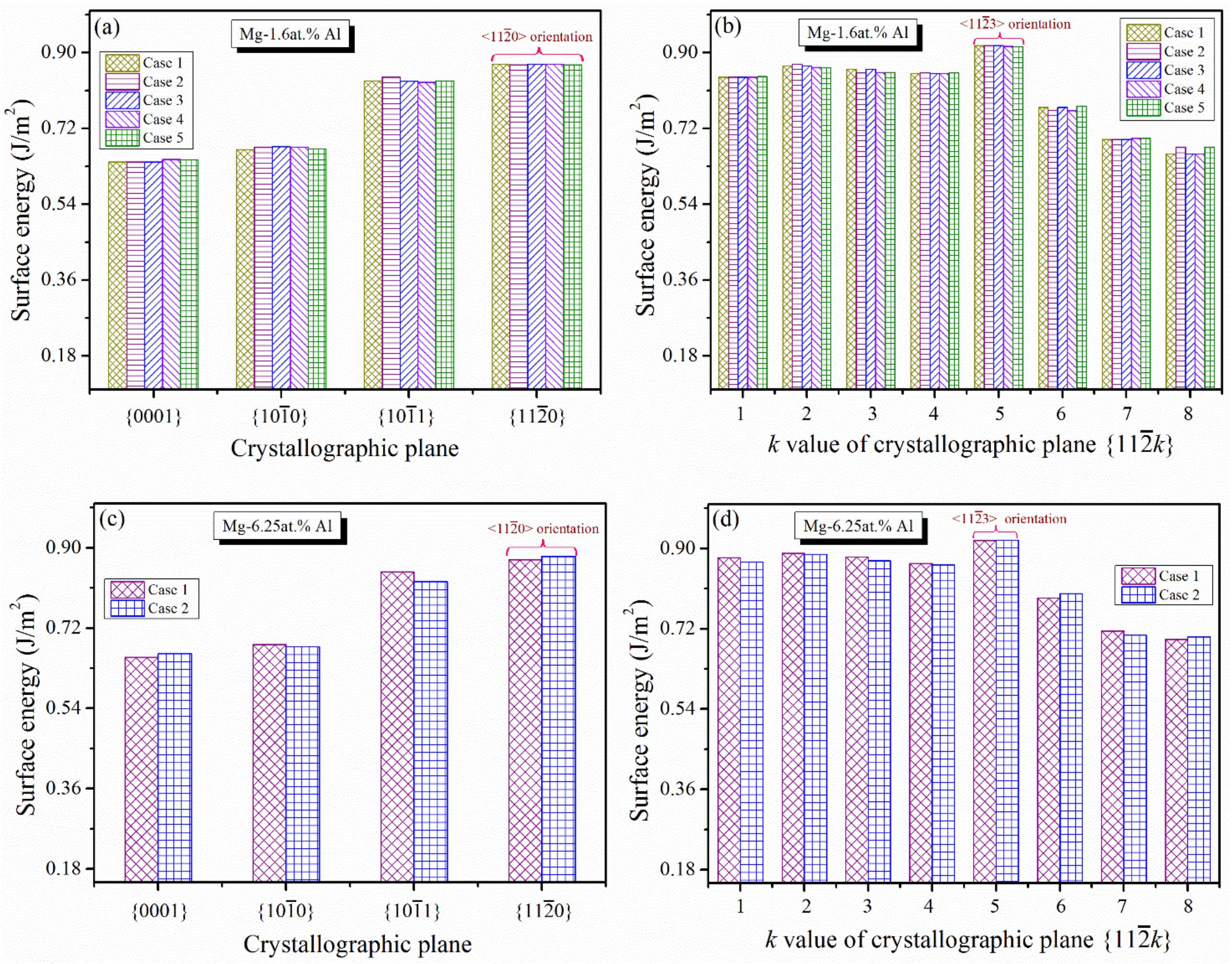

Figure S3. Orientation-dependent surface energy for five cases of Mg-1.6at.%Al alloy and two cases of Mg-6.2at.%Al alloy with different atomic positions of solvent substituted by solute atoms in magnesium 4×4×2 supercell. (a) and (c) show the high symmetrical surface orientations, (b) and (d) show the high index surface orientations, indicating that in any cases, those crystallographic planes (i.e. $\{11\bar{2}0\}$ and $\{11\bar{2}5\}$) corresponding to the preferred growth direction of magnesium alloy dendrite (i.e. $<11\bar{2}0>$ and $<11\bar{2}3>$) have relatively higher surface energy than those of other surface orientations.
